# Safety, Pharmacokinetics, and Pharmacodynamics Evaluation of Ivonescimab, a Novel Bispecific Antibody Targeting PD‐1 and VEGF, in Chinese Patients With Advanced Solid Tumors

**DOI:** 10.1002/cam4.70653

**Published:** 2025-03-20

**Authors:** Fenghua Wang, Xiaoli Wei, Yulong Zheng, Jing Wang, Jieer Ying, Xiaozhong Chen, Suxia Luo, Huiyan Luo, Xufang Yu, Benchao Chen, Lei Ma, Ruihua Xu

**Affiliations:** ^1^ Department of Medical Oncology, Cancer Prevention Center Sun Yat‐Sen University Guangzhou Guangdong China; ^2^ Department of Medical Oncology The First Affiliated Hospital, Zhejiang University School of Medicine Zhejiang Hangzhou China; ^3^ Department of Gynecology Hunan Cancer Hospital Changsha Hunan China; ^4^ Department of Hepatobiliary Pancreatic Gastric Medicine Zhejiang Cancer Hospital Zhejiang Hangzhou China; ^5^ The Department of Head and Neck Radiation Zhejiang Cancer Hospital Zhejiang Hangzhou China; ^6^ Department of Medical Oncology Henan Cancer Hospital Zhengzhou Henan China; ^7^ Akeso Biopharma, Inc. Zhongshan Guangdong China

**Keywords:** advanced solid tumors, AK112, ivonescimab, PD‐1, pharmacodynamics, pharmacokinetics, VEGF

## Abstract

**Background:**

Ivonescimab (AK112) is a first‐in‐class bispecific antibody that simultaneously targets programmed death‐1 (PD‐1) and vascular endothelial growth factor (VEGF) with cooperative binding. We report the safety, pharmacokinetics (PK), and pharmacodynamics (PD) profiles of ivonescimab in patients suffered from advanced solid tumors.

**Methods:**

A multicenter, open‐label, dose‐escalation, phase I study was conducted in five hospitals in China. Ivonescimab was used as a monotherapy. The dose of ivonescimab intravenously administered was 3, 5, 10, 20, and 30 mg/kg every 2 weeks (Q2W), and 10 and 20 mg/kg every 3 weeks (Q3W). Safety, PK, and PD of ivonescimab were evaluated.

**Results:**

A total of 59 patients treated in the study. Only one dose‐limiting toxicity (DLT) occurred in 1 out of 9 patients in the 10 mg/kg Q2W cohort, indicating that no maximum tolerated dose was reached. Among the participants, 53 patients (89.8%) experienced treatment‐related adverse events (TRAEs), with the most common being proteinuria (33.9%), aspartate aminotransferase elevation (27.1%), white blood cell count decrease (22.0%), alanine aminotransferase elevation (20.3%), and anemia (20.3%). Fourteen patients (23.7%) had ≥ Grade 3 TRAEs, and 7 patients (11.9%) experienced serious TRAEs. Notably, there were no reported deaths associated with the TRAEs, and no dose‐dependent increase in adverse events was observed. The half‐life of ivonescimab ranged from 5.0 to 7.3 days following single‐dose administration across all dose levels. The serum concentrations of ivonescimab increased with escalating doses in an approximately dose‐proportional manner. Following multiple doses, the accumulation ratio ranged from 1.1 to 1.7, suggesting mild accumulation of ivonescimab. The steady state was achieved after 5 doses. Ivonescimab occupancy on PD‐1 sustained over 80% across the treatment period. Serum VEGF level was rapidly down‐regulated after each administration.

**Conclusions:**

In patients with advanced solid tumors, ivonescimab monotherapy was well‐tolerated and demonstrated a linear PK characteristics. PD profiles showed the promising potential of ivonescimab for the management of advanced solid tumors.

**Trial Registration:** ClinicalTrials.gov (NCT04597541)

## 1 Introduction

The mechanism of tumor growth is complicated, angiogenesis pathways and immune‐escape pathways were found to be involved [1, 2, 3]. Angiogenesis refers to the growth of new blood vessels from existing ones, and this process is essential to the proliferation and survival of tumor cells. Vascular endothelial growth factor (VEGF) plays a key role in tumor angiogenesis and is overexpressed in many solid tumors [4, 5]. Programmed death‐1 (PD‐1), an inhibitory receptor on activated T cells, suppresses T cells activation by binding with programmed death‐ligand 1 (PD‐L1). Tumor tissue expressing PD‐L1 leads to tumor immune evasion [6]. A combination of anti‐PD‐1/L1 antibody and anti‐VEGF antibody is expected to exert complementary and synergistic anti‐tumor effects.

A number of clinical trials have been developed to explore the clinical benefits of such combinatory approach. A Phase 2 study demonstrates that combining bevacizumab, an anti‐VEGF agent, and nivolumab, a PD‐1 inhibitor, has potential anti‐tumor efficacy in women with relapsed ovarian cancer [7]. Bevacizumab combined with atezolizumab (a PD‐L1 inhibitor) is considered as a first‐line treatment option for untreated metastatic renal cell carcinoma [8, 9, 10]. While the prognosis for non‐small cell lung cancer (NSCLC) treated with anti‐PD‐1 plus platinum‐doublet chemotherapy remains poor, a phase 3 study concludes that adding bevacizumab results in improved outcomes, with the combination of bevacizumab and atezolizumab proving more effective than bevacizumab alone [11]. This combination also shows anti‐tumor activity in hepatocellular carcinoma [12] and metastatic colorectal cancer [13]. These findings strongly indicate that the combination of anti‐PD‐1/L1 and anti‐VEGF therapies represents a promising anti‐tumor strategy.

Ivonescimab (AK112) is a groundbreaking bispecific antibody engineered to specifically target both PD‐1 and VEGF in a cooperative manner such that the binding of each target enhances the binding of the other target. Unlike the traditional approach of combining separate anti‐PD‐1/L1 and anti‐VEGF antibodies, ivonescimab alone can simultaneously achieve anti‐PD‐1 and anti‐VEGF effects, and the ability to cooperatively bind the targets enhances this therapeutic outcome, offering a more efficient and convenient therapeutic option. Besides, VEGF, apart from its role in stimulating tumor angiogenesis, also serves as a key immunosuppressive factor in the tumor microenvironment. It is known that VEGF stimulates PD‐1 expression in tumor‐infiltrating CD8^+^ T cells [14]. The expression of VEGF and PD‐1 is strongly correlated within the tumor microenvironment. Therefore, blocking both PD‐1 and VEGF simultaneously with ivonescimab might offer a more targeted enrichment of drug in the tumor microenvironment.

Based on the preclinical pharmacodynamics and toxicological study data for ivonescimab [15] and the promising evidence of clinical efficacy with manageable safety profile that have been observed for the combination of anti‐PD‐1/L1 and anti‐VEGF drugs across a range of tumor types in early or late‐stage clinical trials, we have sound scientific rationales for clinical investigation of ivonescimab in patients with solid tumors.

The first‐in‐human study of ivonescimab (AK112‐101) was conducted in Australia, and findings on its safety, anti‐tumor activity, pharmacokinetics (PK), and pharmacodynamics (PD) in Australian patients with advanced solid tumors have been recently published [16]. It is recognized that drug responses may be different across ethnic groups. This study (AK112‐102) was therefore conducted to investigate ivonescimab in Chinese patients. A phase II study (AK112‐201) on ivonescimab combined with chemotherapy for advanced NSCLC, a phase Ib/II study (AK112‐202) on ivonescimab as first‐ or second‐line therapy for advanced NSCLC, and a phase III study (AK112‐301) of ivonescimab plus chemotherapy for advanced NSCLC with epidermal growth factor receptor variant were also conducted in China and have been published [17, 18, 19]. However, these publications did not cover PK and PD of ivonescimab. Here we report the phase I study of AK112‐102. Safety, PK, PD, and immunogenicity of ivonescimab in Chinese patients are described.

## 2 Methods

### 2.1 Study Design

AK112‐102 was designed as a multicenter, open‐label, phase I/II study. Phase I was focused on dose‐escalation and phase II was a dose‐expansion phase. The dose‐expansion phase was not undertaken owing to a decision that unrelated to patient safety issues made by the sponsor.

The primary endpoint was safety. The secondary endpoints included anti‐tumor activity (data not shown), PK, and immunogenicity. The exploratory endpoint was PD.

In the dose‐escalation phase, patients received the following doses of ivonescimab: 3, 5, 10, 20, and 30 mg/kg every 2 weeks (Q2W) in a 3 + 3 + 3 design to determine the maximum‐tolerated dose (MTD). Moreover, to explore an alternative dosing schedule of every 3 weeks (Q3W), dose levels of 10 mg/kg Q3W and 20 mg/kg Q3W were also investigated. Each dose level's safety was monitored for 28 days before progressing to the next dose level. If a dose level did not exceed the MTD, it could be expanded to a maximum of 18 subjects. Dose escalation would be terminated if dose‐limiting toxicity (DLT) was observed in ≥ 33% of patients or even if the MTD was not reached by the end of the per‐protocol dose‐escalation phase.

MTD was identified as the highest dose level where < 33% of patients experienced a DLT within the initial 28 days (the DLT observation period). A DLT was defined based on the frequency and severity of treatment‐related adverse events (AEs) during this observation period. The detailed definitions of DLTs evaluated to be related to ivonescimab are listed in Table S1.

### 2.2 Study Treatment

Ivonescimab was used as a monotherapy in the dose‐escalation phase. It was administered via intravenous infusion over a period of 60–120 min (± 10 min) on day 1 and day 15 of each 28‐day treatment cycle for Q2W cohorts, and on day 1 of each 21‐day treatment cycle for Q3W cohorts. The administration of ivonescimab continued until a maximum of 24 months, the development of unacceptable toxicity, radiographic disease progression, death, loss to follow‐up, sponsor termination of the study, subject withdrawal of consent, or other termination criteria in the protocol were met. The safety follow‐up period was 30 days after the end of treatment. Patients were followed up every 12 weeks for survival.

### 2.3 Patients

AK112‐102 was conducted at five sites in China. Approval was obtained from independent ethics committee at each site. Prior to enrolling in the study, all patients provided their written informed consent. The study was performed in accordance with the Good Clinical Practice guidelines and the Declaration of Helsinki. The study is registered on ClinicalTrials.gov (NCT04597541).

Eligible patients participating in the study were 18–75 years old and had histologically or cytologically confirmed advanced or metastatic solid tumors. These patients have experienced treatment failure with at least one prior standard therapy, or had no effective standard therapy available, or were unable to tolerate or refused to receive standard therapy. Patients were required to have at least one measurable lesion for tumor assessment. A performance status of 0 or 1 according to Eastern Cooperative Oncology Group performance status (ECOG PS), a life expectancy of minimum 12 weeks, and satisfactory bone marrow, liver, and renal function were also required.

Patients were excluded if they reported known severe hypersensitivity reactions to other monoclonal or bispecific antibodies; active malignancy within the past 5 years except for the tumor for which a subject was enrolled in the study and locally curable cancers that have been apparently cured; concurrent enrollment in another clinical study; receipt of systemic anti‐tumor therapy within 3 weeks or treatment with small‐molecule tyrosine kinase inhibitor within 2 weeks before the first dose of investigational product; known brainstem or leptomeningeal metastases, or spinal cord metastasis or compression, or active central nervous system metastases; pleural effusion, pericardial effusion, or ascites with clinical symptoms or requiring repeated drainage. Patients were also excluded if the imaging at screening showed that tumors surrounded important blood vessels or had obvious necrosis or cavities, and the investigator determined that participation in the study would cause a risk of bleeding during the study period. Additional exclusion criteria included history and/or current evidence of gastrointestinal perforation, surgical and wound healing complications, and bleeding events; current evidence of clinically significant cardiac disease or uncontrolled diseases; receipt of any PD‐1/PD‐L1/CTLA‐4 inhibitors or any immunotherapy ever. The full inclusion and exclusion criteria are outlined in Table S2.

### 2.4 Assessments

Safety was assessed through AEs, lab values, physical exams, vital signs, electrocardiogram, and anti‐drug antibodies (ADAs) positivity. A treatment‐emergent AE (TEAE) referred to an AE that either newly appeared or worsening from the first dose of investigational product up to the safety follow‐up. A treatment‐related AE (TRAE) was defined as a TEAE that was related to or possibly related to the investigational product as per the investigator, or a TEAE with missing assessment of the causality. Severity of AEs was graded according to the National Cancer Institute Common Terminology Criteria for Adverse Events (NCI CTCAE) version 5.0.

ADA blood samples were collected before ivonescimab infusion on day 1 of Cycles 1, 2, 3, 4, 6, 8, 10, 12, and every 2 cycles thereafter; as well as 30 and 90 days after the last ivonescimab infusion. ADAs were measured using an electrochemiluminescent immunoassay using Meso Scale Discovery technology (Rockville, Maryland, USA). A screening assay was followed by a confirmatory assay. If both assays returned a positive result, the sample was categorized as ADA positive and the titer evaluation was applied. Samples that presented treatment‐emergent ADA positive were further assayed for their neutralizing activity.

PK and PD blood samples were obtained at specified time points detailed in Tables S3 and S4, respectively. Serum concentrations of ivonescimab for PK analysis were determined by a validated enzyme‐linked immunosorbent assay (ELISA) with a lower limit of quantification of 0.01 μg/mL. PD assessments included receptor occupancy (RO) on PD‐1 of peripheral blood CD3+ T cells and serum level of VEGF. RO was tested by qualified flow cytometry. Serum concentrations of VEGF were measured by ELISA with a lower limit of quantification of 15.6 pg/mL.

### 2.5 Statistical Analysis

No formal sample size estimation was performed. Fifty‐four patients were planned for enrollment. Demographics and baseline characteristics, safety, drug concentration, PK, and PD were presented as descriptive statistics. PK parameters were estimated using non‐compartmental analysis by Phoenix WinNonlin software (version 8.2 or above; Certara USA Inc., Princeton, New Jersey, USA). The definitions of PK parameters can be found in Table S5. Dose proportionality in Q2W cohorts was evaluated using a power model on log‐transformed PK parameters relative to log‐transformed dose, which is described as log(parameter) = loga + β × log(dose) + error, where a represents the intercept and β represents the slope. All the statistical analyses were performed using SAS software (version 9.4 or above; SAS Institute Inc., Cary, North Carolina, USA).

## 3 Results

### 3.1 Patients

**FIGURE 1:**
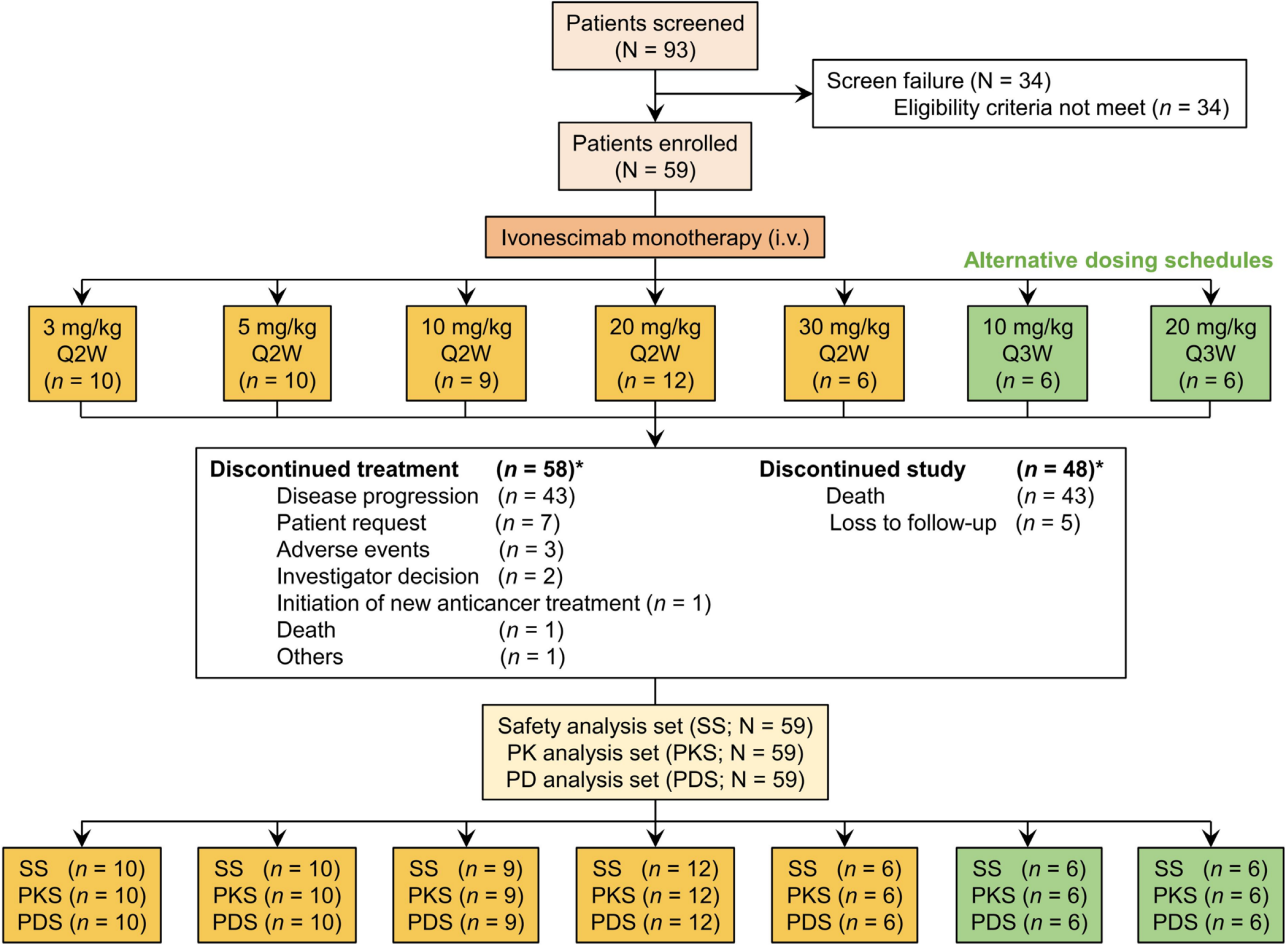
Study flow and patient disposition. Patients who received at least one dose of ivonescimab were included in SS; patients who had at least one valid on‐treatment PK variable were included in PKS; patients with baseline and at least one evaluable post‐baseline PD data were included in PDS. i.v., intravenous; Q2W, every 2 weeks; Q3W, every 3 weeks; SS, safety analysis set; PK, pharmacokinetic; PKS, PK analysis set; PD, pharmacodynamic; PDS, PD analysis set. *Patient disposition at the time of data cutoff.

Among 93 patients screened, a total of 59 patients were enrolled and received treatment with ivonescimab (Figure 1). The median age was 55 years (range: 24–73 years) and the majority were female (59.3%) (Table 1). Forty‐three patients (72.9%) had an ECOG PS of 1. The most common tumor types were colorectal cancer (*n* = 10, 16.9%), epithelial ovarian cancer (*n* = 6, 10.2%), hepatocellular carcinoma (*n* = 6, 10.2%), cervical cancer (*n* = 6, 10.2%), and breast cancer (*n* = 5, 8.5%). The median number of previous systemic therapy lines for metastatic disease was two (range: 0–6), with 39% of patients having received at least three systemic therapy lines for metastatic disease. Fifty‐eight patients (98.3%) received prior chemotherapy, 19 patients (32.2%) received prior anti‐PD‐1 immunotherapy, 39 patients (66.1%) received prior VEGF‐targeted therapy, and 14 patients (23.7%) received prior anti‐PD‐L1 plus anti‐VEGF therapy.

**TABLE 1:** Baseline characteristics of patients.

Characteristic	3 mg/kg Q2W (*n* = 10)	5 mg/kg Q2W (*n* = 10)	10 mg/kg Q2W (*n* = 9)	20 mg/kg Q2W (*n* = 12)	30 mg/kg Q2W (*n* = 6)	10 mg/kg Q3W (*n* = 6)	20 mg/kg Q3W (*n* = 6)	Total (*n* = 59)
**Age (years)**								
Median	54.0	51.5	57.0	59.5	47.5	56.0	54.5	55.0
Min, Max	37.0, 68.0	35.0, 68.0	52.0, 67.0	42.0, 73.0	24.0, 59.0	51.0, 58.0	28.0, 73.0	24.0, 73.0
≤ 65, *n* (%)	9 (90.0)	8 (80.0)	8 (88.9)	9 (75.0)	6 (100.0)	6 (100.0)	5 (83.3)	51 (86.4)
> 65, *n* (%)	1 (10.0)	2 (20.0)	1 (11.1)	3 (25.0)	0	0	1 (16.7)	8 (13.6)
**Sex, *n* (%)**								
Male	6 (60.0)	1 (10.0)	3 (33.3)	5 (41.7)	4 (66.7)	2 (33.3)	3 (50.0)	24 (40.7)
Female	4 (40.0)	9 (90.0)	6 (66.7)	7 (58.3)	2 (33.3)	4 (66.7)	3 (50.0)	35 (59.3)
**Race, *n* (%)**								
Asian	10 (100.0)	10 (100.0)	9 (100.0)	12 (100.0)	6 (100.0)	6 (100.0)	6 (100.0)	59 (100.0)
**Weight (kg)**								
Median	60.0	50.5	58.0	56.4	56.0	57.9	62.7	56.0
Min, Max	49.6, 85.4	45.4, 68.0	43.0, 78.2	42.0, 68.8	45.0, 88.3	36.2, 81.2	46.6, 80.2	36.2, 88.3
**BMI (kg/m** ^**2**^)								
Median	23.5	20.6	22.3	22.3	20.3	22.2	24.0	22.2
Min, Max	19.9, 28.7	19.1, 28.7	19.2, 29.4	16.1, 26.3	18.3, 31.3	16.5, 28.8	17.9, 32.3	16.1, 32.3
**ECOG PS, *n* (%)**								
0	6 (60.0)	1 (10.0)	4 (44.4)	2 (16.7)	2 (33.3)	1 (16.7)	0	16 (27.1)
1	4 (40.0)	9 (90.0)	5 (55.6)	10 (83.3)	4 (66.7)	5 (83.3)	6 (100.0)	43 (72.9)
**Tumor histology, *n* (%)**								
Epithelial ovarian	1 (10.0)	3 (30.0)	1 (11.1)	1 (8.3)	0	0	0	6 (10.2)
Non‐small cell lung	0	1 (10.0)	0	0	0	0	0	1 (1.7)
Hepatocellular	0	0	1 (11.1)	1 (8.3)	2 (33.3)	2 (33.3)	0	6 (10.2)
Colorectal	6 (60.0)	1 (10.0)	3 (33.3)	0	0	0	0	10 (16.9)
Gastric	0	0	1 (11.1)	2 (16.7)	1 (16.7)	0	0	4 (6.8)
Renal cell	0	0	0	0	1 (16.7)	0	2 (33.3)	3 (5.1)
Small cell lung	0	0	1 (11.1)	0	0	0	2 (33.3)	3 (5.1)
Cervical	2 (20.0)	2 (20.0)	1 (11.1)	0	1 (16.7)	0	0	6 (10.2)
Esophageal	0	1 (10.0)	0	0	0	0	0	1 (1.7)
Breast	0	0	0	1 (8.3)	0	3 (50.0)	1 (16.7)	5 (8.5)
Endometrial	0	1 (10.0)	1 (11.1)	0	0	0	0	2 (3.4)
Others	1 (10.0)	1 (10.0)	0	7 (58.3)	1 (16.7)	1 (16.7)	1 (16.7)	12 (20.3)
**Lines of prior chemotherapy, *n* (%)**								
0	0	0	0	1 (8.3)	0	0	0	1 (1.7)
1	1 (10.0)	2 (20.0)	2 (22.2)	6 (50.0)	2 (33.3)	0	1 (16.7)	14 (23.7)
2	3 (30.0)	5 (50.0)	2 (22.2)	2 (16.7)	4 (66.7)	3 (50.0)	2 (33.3)	21 (35.6)
3	2 (20.0)	2 (20.0)	4 (44.4)	3 (25.0)	0	2 (33.3)	2 (33.3)	15 (25.4)
4	2 (20.0)	1 (10.0)	0	0	0	1 (16.7)	1 (16.7)	5 (8.5)
5	1 (10.0)	0	1 (11.1)	0	0	0	0	2 (3.4)
6	1 (10.0)	0	0	0	0	0	0	1 (1.7)

Abbreviations: BMI, body mass index; ECOG PS, Eastern Cooperative Oncology Group performance status; Q2W, every 2 weeks; Q3W, every 3 weeks.

As of data cutoff on July 30, 2023, the median follow‐up was 24.1 months (range: 1.7–30.3 months). Ivonescimab treatment was still ongoing for one of 6 patients in the 30 mg/kg Q2W cohort. The median duration of exposure to ivonescimab for all patients was 113 days (range: 14–716 days) and the median treatment frequency was 5 times (range: 1–36 times). Table S8 summarizes the duration of exposure to ivonescimab in each dosage cohort.

### 3.2 Safety

One DLT occurred in one patient (< 33%) receiving one dose of 10 mg/kg Q2W ivonescimab. The patient developed immune‐mediated cholangitis (CTCAE Grade 3) on Cycle 1 day 8, which the investigator deemed possibly related to ivonescimab. This patient discontinued ivonescimab treatment thereafter. No other DLTs were observed. Therefore, the MTD of ivonescimab was not identified within the 3–30 mg/kg dose range.

AEs are summarized in Table 2. Among 59 patients, 56 patients (94.9%) experienced at least one TEAE, with 53 patients (89.8%) experiencing at least one TRAE. TRAEs that occurred in ≥ 20% of patients were proteinuria (33.9%), aspartate aminotransferase increase (27.1%), white blood cell count decrease (22.0%), alanine aminotransferase increase (20.3%), and anemia (20.3%). Grade 3 or higher TRAEs were reported in 14 patients (23.7%), with hypertension (5.1%) being the most common (≥ 5% frequency). Serious TRAEs were reported in 7 patients (11.9%). Two patients (3.4%) discontinued treatment due to a TRAE, which included Grade 2 immune‐mediated pneumonitis and Grade 3 immune‐mediated cholangitis. No deaths were reported among all the TRAEs. In addition, a consistent safety profile was found across the 7 dose cohorts.

**TABLE 2:** Summary of adverse events.

Adverse events	3 mg/kg Q2W (*n* = 10) *n* (%)	5 mg/kg Q2W (*n* = 10) *n* (%)	10 mg/kg Q2W (*n* = 9) *n* (%)	20 mg/kg Q2W (*n* = 12) *n* (%)	30 mg/kg Q2W (*n* = 6) *n* (%)	10 mg/kg Q3W (*n* = 6) *n* (%)	20 mg/kg Q3W (*n* = 6) *n* (%)	Total (*n* = 59) *n* (%)
**Any TEAEs**	10 (100.0)	9 (90.0)	9 (100.0)	12 (100.0)	6 (100.0)	4 (66.7)	6 (100.0)	56 (94.9)
**≥ Grade 3 TEAEs**	4 (40.0)	4 (40.0)	5 (55.6)	9 (75.0)	3 (50.0)	2 (33.3)	2 (33.3)	29 (49.2)
**Serious TEAEs**	3 (30.0)	5 (50.0)	4 (44.4)	8 (66.7)	2 (33.3)	2 (33.3)	0	24 (40.7)
**TEAEs leading to treatment suspension**	1 (10.0)	6 (60.0)	2 (22.2)	7 (58.3)	3 (50.0)	1 (16.7)	2 (33.3)	22 (37.3)
**TEAEs leading to treatment discontinuation**	0	2 (20.0)	1 (11.1)	0	0	0	0	3 (5.1)
**TEAEs leading to death**	3 (30.0)	2 (20.0)	3 (33.3)	3 (25.0)	1 (16.7)	1 (16.7)	0	13 (22.0)
**Any TRAEs**	$10\;\left(100.0\right)$	$9\;\left(90.0\right)$	$9\;\left(100.0\right)$	$10\;\left(83.3\right)$	$5\;\left(83.3\right)$	$4\;\left(66.7\right)$	$6\;\left(100.0\right)$	$53\;\left(89.8\right)$
**Any TRAEs with a frequency of ≥ 10% in total patients**								
Aspartate aminotransferase elevation	$3\;\left(30.0\right)$	$1\;\left(10.0\right)$	$1\;\left(11.1\right)$	$3\;\left(25.0\right)$	$3\;\left(50.0\right)$	$1\;\left(16.7\right)$	$4\;\left(66.7\right)$	$16\;\left(27.1\right)$
White blood cell count decrease	$2\;\left(20.0\right)$	$2\;\left(20.0\right)$	$2\;\left(22.2\right)$	$4\;\left(33.3\right)$	$2\;\left(33.3\right)$	$1\;\left(16.7\right)$	0	$13\;\left(22.0\right)$
Alanine aminotransferase elevation	$2\;\left(20.0\right)$	0	$1\;\left(11.1\right)$	$2\;\left(16.7\right)$	$3\;\left(50.0\right)$	$1\;\left(16.7\right)$	$3\;\left(50.0\right)$	$12\;\left(20.3\right)$
Platelet count decrease	$1\;\left(10.0\right)$	$1\;\left(10.0\right)$	0	$5\;\left(41.7\right)$	$1\;\left(16.7\right)$	$1\;\left(16.7\right)$	$1\;\left(16.7\right)$	$10\;\left(16.9\right)$
Gamma−glutamyltransferase elevation	0	0	$1\;\left(11.1\right)$	$5\;\left(41.7\right)$	$1\;\left(16.7\right)$	$2\;\left(33.3\right)$	0	$9\;\left(15.3\right)$
Blood pressure elevation	$3\;\left(30.0\right)$	0	$3\;\left(33.3\right)$	0	0	0	0	$6\;\left(10.2\right)$
Weight decrease	$1\;\left(10.0\right)$	$2\;\left(20.0\right)$	$2\;\left(22.2\right)$	0	$1\;\left(16.7\right)$	0	0	$6\;\left(10.2\right)$
Proteinuria	$3\;\left(30.0\right)$	$3\;\left(30.0\right)$	$2\;\left(22.2\right)$	$6\;\left(50.0\right)$	$3\;\left(50.0\right)$	$1\;\left(16.7\right)$	$2\;\left(33.3\right)$	$20\;\left(33.9\right)$
Hypoalbuminaemia	$1\;\left(10.0\right)$	0	$1\;\left(11.1\right)$	$4\;\left(33.3\right)$	$1\;\left(16.7\right)$	0	$1\;\left(16.7\right)$	$8\;\left(13.6\right)$
Decreased appetite	$1\;\left(10.0\right)$	$2\;\left(20.0\right)$	$1\;\left(11.1\right)$	0	$3\;\left(50.0\right)$	0	0	$7\;\left(11.9\right)$
Anemia	$1\;\left(10.0\right)$	$2\;\left(20.0\right)$	$1\;\left(11.1\right)$	$3\;\left(25.0\right)$	$3\;\left(50.0\right)$	$2\;\left(33.3\right)$	0	$12\;\left(20.3\right)$
Hypothyroidism	$1\;\left(10.0\right)$	$2\;\left(20.0\right)$	$1\;\left(11.1\right)$	$1\;\left(8.3\right)$	0	0	$1\;\left(16.7\right)$	$6\;\left(10.2\right)$
Hypertension	0	0	0	$3\;\left(25.0\right)$	$2\;\left(33.3\right)$	0	$1\;\left(16.7\right)$	$6\;\left(10.2\right)$
**≥ Grade 3 TRAEs**	$3\;\left(30.0\right)$	$1\;\left(10.0\right)$	$2\;\left(22.2\right)$	$3\;\left(25.0\right)$	$2\;\left(33.3\right)$	$1\;\left(16.7\right)$	$2\;\left(33.3\right)$	$14\;\left(23.7\right)$
**≥ Grade 3 TRAEs with a frequency of** ≥ **3% in total patients**								
Gamma‐glutamyltransferase elevation	0	0	$1\;\left(11.1\right)$	0	0	$1\;\left(16.7\right)$	0	$2\;\left(3.4\right)$
Blood pressure elevation	$2\;\left(20.0\right)$	0	0	0	0	0	0	$2\;\left(3.4\right)$
Hypertension	0	0	0	$1\;\left(8.3\right)$	$1\;\left(16.7\right)$	0	$1\;\left(16.7\right)$	$3\;\left(5.1\right)$
Proteinuria	0	0	0	0	$2\;\left(33.3\right)$	0	0	$2\;\left(3.4\right)$
**Serious TRAEs**	$2\;\left(20.0\right)$	$1\;\left(10.0\right)$	$1\;\left(11.1\right)$	$2\;\left(16.7\right)$	0	$1\;\left(16.7\right)$	0	$7\;\left(11.9\right)$
**TRAEs leading to treatment suspension**	$1\;\left(10.0\right)$	$6\;\left(60.0\right)$	0	$4\;\left(33.3\right)$	$2\;\left(33.3\right)$	$1\;\left(16.7\right)$	$2\;\left(33.3\right)$	$16\;\left(27.1\right)$
**TRAEs leading to treatment discontinuation**	0	$1\;\left(10.0\right)$	$1\;\left(11.1\right)$	0	0	0	0	$2\;\left(3.4\right)$
**TRAEs leading to death**	0	0	0	0	0	0	0	0

Abbreviations: Q2W, every 2 weeks; Q3W, every 3 weeks; TEAEs, treatment‐emergent adverse events; TRAEs, treatment‐related adverse events.

The immunogenicity analysis excluded 4 patients because post‐baseline ADA results were not available for them. Table S6 summarizes the incidence of immunogenicity in 55 patients. Post‐baseline ADAs were detected in 8 out of 55 patients (14.5%). Among them, 5 patients (9.1%) developed non‐treatment‐emergent ADAs, while treatment‐emergent ADAs were found in 3 patients (5.5%) from the 3 mg/kg Q2W (*n* = 2) and 5 mg/kg Q2W (*n* = 1) cohorts. Of these 3 patients, 2 from the 3 mg/kg Q2W cohort developed anti‐PD‐1 neutralizing antibodies, whereas all of them developed anti‐VEGF neutralizing antibodies.

### 3.3 Pharmacokinetics

**FIGURE 2:**
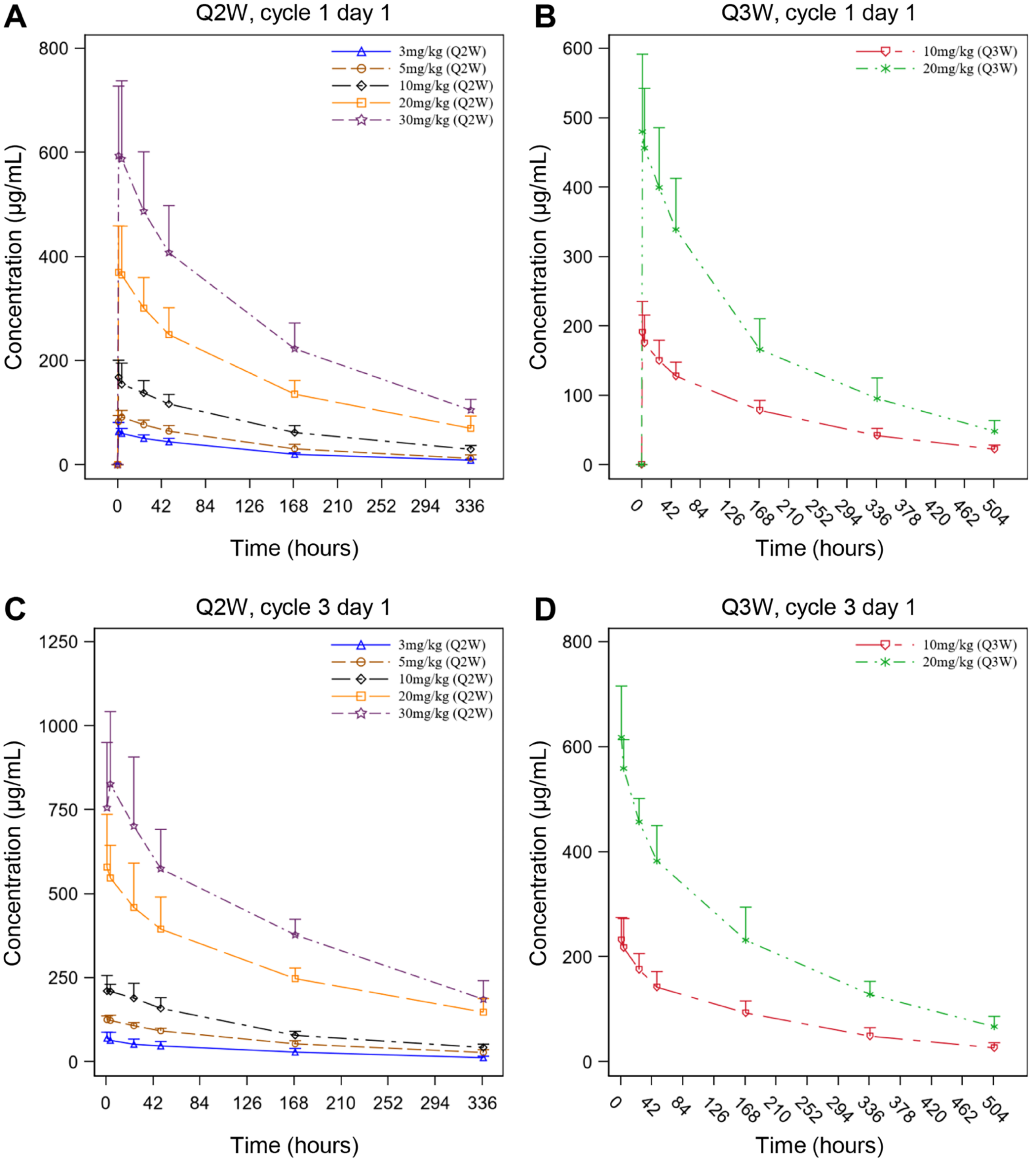
Serum concentrations of ivonescimab following single‐dose and multiple‐dose intravenous administrations. The serum concentration‐time curves of ivonescimab in (A) Q2W and (B) Q3W cohorts after a single dose, and the serum concentration‐time curves of ivonescimab in (C) Q2W and (D) Q3W cohorts after multiple doses were displayed. Data were expressed as mean + SD. Q2W, every 2 weeks; Q3W, every 3 weeks.

The serum concentration‐time curves of ivonescimab following a single dose are presented in Figure 2A,B, showing an increase in serum concentrations with doses ranging from 3 to 30 mg/kg. Table 3 summarizes the PK parameters, including median time to reach the maximum concentration (T_max_), mean half‐life (t_1/2_), and mean clearance (CL) across all dose cohorts, falling within the ranges of 1.10–3.95 h, 4.98–7.30 days, and 0.328–0.491 L/day, respectively. There was no apparent dose‐dependence in T_max_, t_1/2_, and CL. Both maximum concentration (C_max_) and area under the concentration‐time curve (AUC) increased in a dose‐related manner in the range of 3 to 30 mg/kg (Figures S1A,B). The slopes (β) for C_max_, AUC_0‐t_, and AUC_0‐∞_ were 0.96 (90% confidence interval [CI], 0.90–1.02), 0.99 (90% CI, 0.92–1.05), and 1.04 (90% CI, 0.97–1.11), respectively (Table 4).

**TABLE 3:** Pharmacokinetic parameters of ivonescimab in serum following single‐dose and multiple‐dose intravenous administrations.

Parameter	3 mg/kg Q2W (*n* = 10)	5 mg/kg Q2W (*n* = 10)	10 mg/kg Q2W (*n* = 9)	20 mg/kg Q2W (*n* = 12)	30 mg/kg Q2W (*n* = 6)	10 mg/kg Q3W (*n* = 6)	20 mg/kg Q3W (*n* = 6)
**After the first dose on Cycle 1 Day 1**							
*n*	10	10	9	12	6	6	6
T_max_ (h)	3.90 (1.00, 4.00)	3.95 (1.00, 4.10)	1.10 (1.00, 4.10)	3.00 (1.00, 4.00)	1.55 (1.00, 5.00)	1.60 (1.00, 4.50)	2.05 (1.00, 24.4)
C_max_ (μg/mL)	64.1 (17.2)	92.7 (12.2)	173 (30.4)	382 (93.6)	600 (139)	193 (44.1)	488 (104)
AUC_0‐t_ (day·μg/mL)	355 (42.9)	545 (104)	999 (205)	2238 (552)	3724 (672)	1448 (239)	3453 (696)
AUC_0‐∞_ (day·μg/mL)	417 (51.0)	644 (165)	1259 (269)	2907 (734)	4685 (788)	1686 (300)	3931 (833)
t_1/2_ (day)	5.12 (0.601)	4.98 (1.62)	5.82 (1.20)	6.13 (0.999)	6.33 (0.915)	7.30 (1.19)	7.16 (1.00)
V_z_ (L)	3.34 (0.513)	2.89 (0.523)	4.05 (1.08)	3.49 (0.623)	3.53 (0.742)	3.55 (0.318)	3.43 (0.96)
CL (L/day)	0.456 (0.0789)	0.433 (0.117)	0.491 (0.126)	0.406 (0.101)	0.393 (0.102)	0.346 (0.0690)	0.328 (0.0505)
**After the fifth/third dose on Cycle 3 Day 1**							
*n*	5	5	5	5	5	5	5
T_max,ss_ (h)	1.10 (1.00, 4.10)	4.10 (1.00, 4.30)	4.00 (1.00, 4.20)	1.10 (1.00, 5.00)	4.60 (1.10, 23.9)	1.90 (1.00, 4.00)	1.00 (1.00, 2.00)
C_max,ss_ (μg/mL)	67.4 (25.5)	126 (12.6)	220 (35.1)	602 (145)	841 (212)	239 (47.3)	618 (97.6)
C_min,ss_ (μg/mL)	10.3 (4.87)	25.6 (8.58)	36.8 (5.35)	150 (49.9)	183 (71.3)	24.2 (9.49)	58.0 (20.4)
C_avg_ (μg/mL)	30.5 (10.0)	61.2 (8.53)	102 (20.1)	262 (71.9)	395 (82.9)^a^	75.2 (17.6)	212 (31.8)
AUC_0‐τ,ss_ (day·μg/mL)	427 (140)	857 (119)	1422 (282)	3665 (1007)	5530 (1160)^a^	1580 (370)	4446 (668)
t_1/2,ss_ (day)	6.19 (0.812)	7.29 (1.63)	5.88 (0.693)	6.86 (2.30)	7.53 (0.389)^a^	6.68 (1.85)	9.08 (2.42)
V_ss_ (L)	3.61 (0.615)	3.29 (1.01)	3.25 (0.976)	3.15 (0.697)	3.70 (1.10)^a^	3.38 (0.546)	4.12 (1.90)
CL_ss_ (L/day)	0.414 (0.107)	0.312 (0.0418)	0.381 (0.0991)	0.349 (0.153)	0.340 (0.0994)^a^	0.363 (0.0591)	0.307 (0.0689)
R_ac,AUC0‐τ_	1.15 (0.359)	1.55 (0.178)	1.48 (0.113)	1.44 (0.340)	1.68 (0.316)^a^	1.13 (0.160)	1.22 (0.138)
R_ac,Cmax_	1.16 (0.520)	1.35 (0.219)	1.34 (0.211)	1.48 (0.363)	1.49 (0.282)	1.26 (0.146)	1.20 (0.125)

*Note:* T_max_ and T_max,ss_ were expressed as median (min, max) and other parameters were expressed as mean (SD).

Abbreviations: AUC_0‐∞_, the area under the concentration‐time curve from 0 to infinity; AUC_0‐t_, the area under the concentration‐time curve from 0 to t days; AUC_0‐τ,ss_, the area under the concentration‐time curve during a dosing interval at steady state; C_avg_, the average concentration at steady state; CL, the clearance; CL_ss_, the clearance at steady state; C_max_, the maximum concentration; C_max,ss_, the maximum concentration at steady state; C_min,ss_, the minimum concentration at steady state; Q2W, every 2 weeks; Q3W, every 3 weeks.R_ac,AUC0‐τ_, the accumulation ratio of AUC_0‐τ_; R_ac,Cmax_, the accumulation ratio of C_max_; t_1/2_, the terminal elimination half‐life; t_1/2,ss_, the terminal elimination half‐life at steady state; T_max_, the time to reach the maximum concentration; T_max,ss_, the time to reach the maximum concentration at steady state; V_ss_, the volume of distribution at steady state; V_z_, the volume of distribution.

^a^ *n* = 4.

**TABLE 4:** Dose proportionality assessment for pharmacokinetic parameters of ivonescimab.

Parameter	Dose range (mg/kg)	Estimate (β)	90% confidence interval
**After a single dose**			
C_max_	3–30	0.96	[0.90, 1.02]
AUC_0‐t_	3–30	0.99	[0.92, 1.05]
AUC_0‐∞_	3–30	1.04	[0.97, 1.11]
**After multiple doses**			
C_max,ss_	3–30	1.06	[0.97, 1.16]
AUC_0‐τ,ss_	3–30	1.03	[0.92, 1.14]

Abbreviations: AUC_0‐∞_, the area under the concentration‐time curve from 0 to infinity; AUC_0‐t_, the area under the concentration‐time curve from 0 to t days; AUC_0‐τ,ss_, the area under the concentration‐time curve during a dosing interval at steady state; C_max_, the maximum concentration; C_max,ss_, the maximum concentration at steady state.

After multiple dosing, a similar PK profile was observed (Figure 2C,D). The median *T*_max,ss_ ranged from 1.00 to 4.60 h and the mean CL_ss_ ranged from 0.307 to 0.414 L/day (Table 3). PK parameters such as *T*_max,ss_, *t*_1/2,ss_, and CL_ss_ were comparable among different dose levels. C_max,ss_ and AUC_0‐τ,ss_ showed a dose‐proportional increase in the 3 to 30 mg/kg range (Figure S1C,D). The slopes (β) for *C*_max,ss_ and AUC_0‐τ,ss_ were 1.06 (90% CI, 0.97–1.16) and 1.03 (90% CI, 0.92–1.14), respectively (Table 4). Upon multiple dosing, the mean accumulation ratios of C_max_ and AUC, namely *R*_ac,Cmax_ and *R*_ac,AUC0‐τ_, ranged between 1.16–1.49 and 1.13–1.68, respectively, suggesting a mild accumulation of ivonescimab exposure at steady state (Table 3).

**FIGURE 3:**
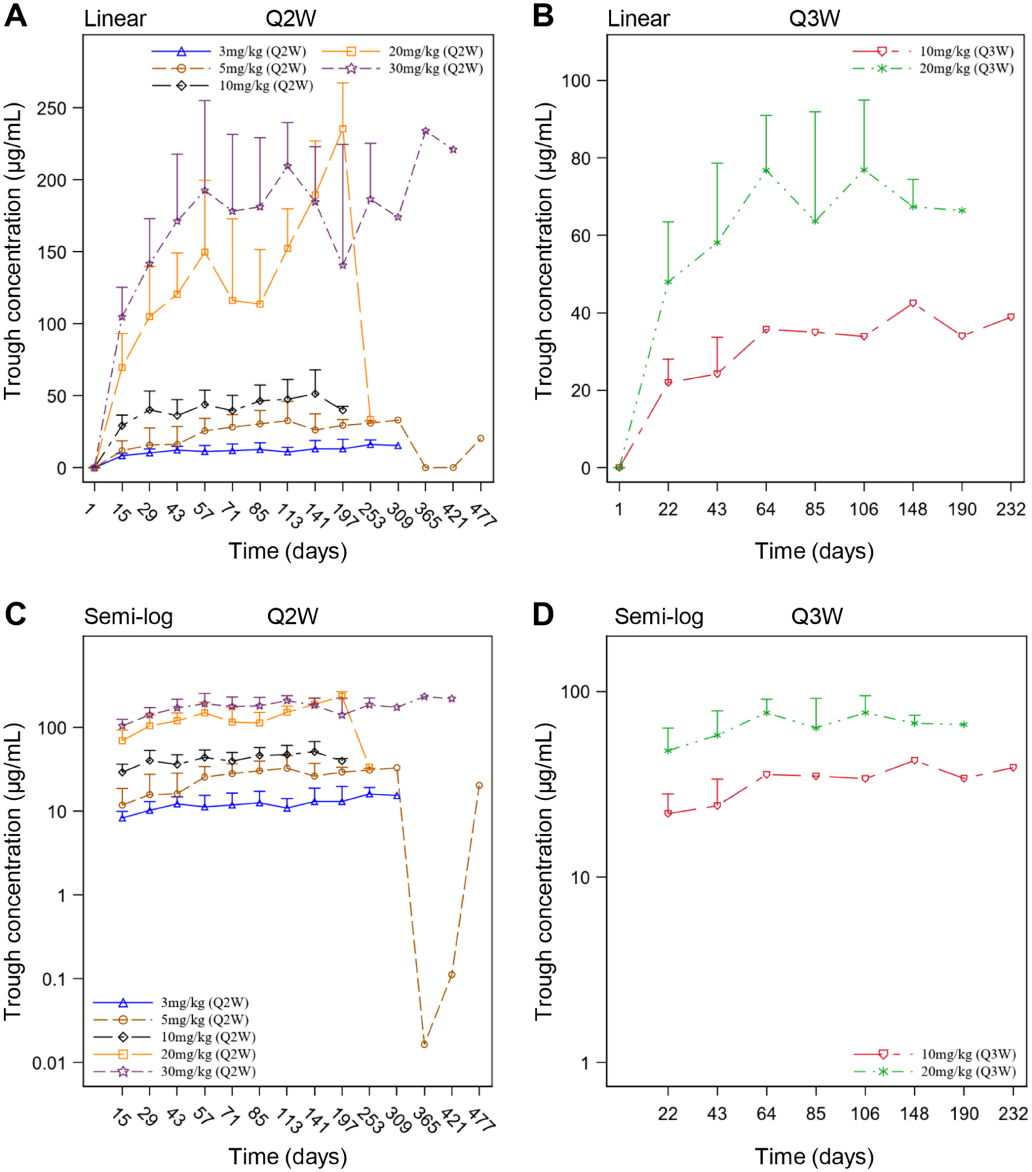
Serum trough concentrations of ivonescimab following multiple‐dose intravenous administration. Linear plots of serum trough concentration‐time profiles of ivonescimab in (A) Q2W and (B) Q3W cohorts after multiple doses were depicted. Semi‐logarithmic plots of serum trough concentration‐time profiles of ivonescimab in (C) Q2W and (D) Q3W cohorts after multiple doses were depicted. Data were expressed as mean + SD. Q2W, every 2 weeks; Q3W, every 3 weeks; semi‐log, semi‐logarithmic.

The serum trough concentrations of ivonescimab following multiple doses are listed in Table S7 and the serum trough concentration‐time curves are displayed in Figure 3. The findings revealed a relatively constant mean trough concentration of ivonescimab after approximately 5 doses.

### 3.4 Pharmacodynamics

**FIGURE 4:**
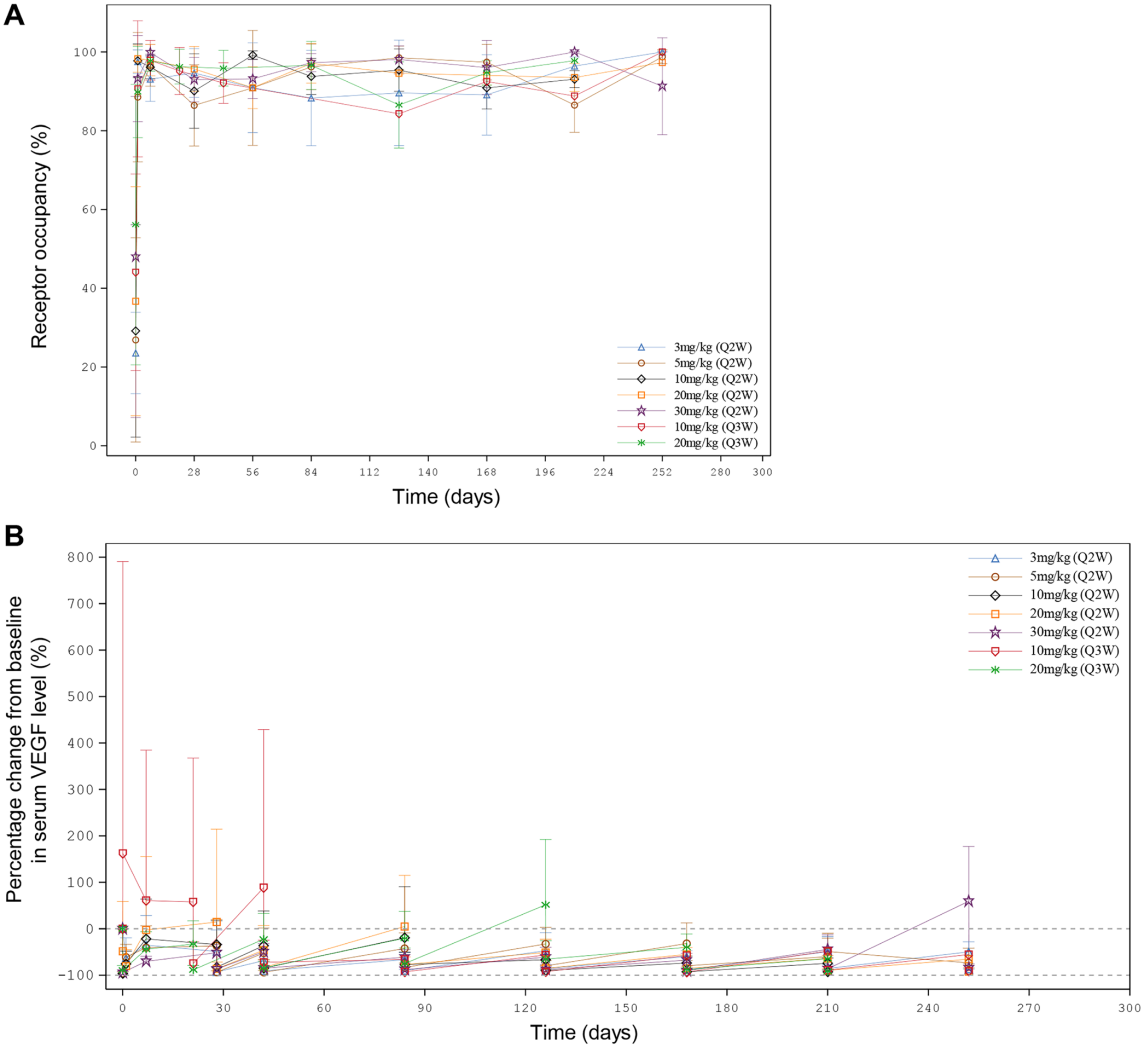
Pharmacodynamics of ivonescimab following multiple‐dose intravenous administration. (A) Receptor occupancy on PD‐1. Data were expressed as mean ± SD. (B) Percentage change from baseline in serum VEGF level. Data were expressed as mean + SD. Q2W, every 2 weeks; Q3W, every 3 weeks; VEGF, vascular endothelial growth factor; PD‐1, programmed death‐1.

The results of PD analyses are presented in Figure 4 and Table 5. For all dose cohorts, the mean ivonescimab occupancy on PD‐1 rapidly increased to approximately 90% 1 day after the first dose, and maintained over 80% throughout the treatment period (Figure 4A and Table 5).

**TABLE 5:** Pharmacodynamic parameters of ivonescimab following multiple‐dose intravenous administration.

Sampling time point	3 mg/kg Q2W (*n* = 10)	5 mg/kg Q2W (*n* = 10)	10 mg/kg Q2W (*n* = 9)	20 mg/kg Q2W (*n* = 12)	30 mg/kg Q2W (*n* = 6)	10 mg/kg Q3W (*n* = 6)	20 mg/kg Q3W (*n* = 6)
**Receptor occupancy on PD‐1 (%)**							
Day 1 predose	23.6 (10.3)	26.9 (25.9)	29.2 (27.0)	36.7 (29.1)	47.9 (40.7)	44.1 (24.9)	56.2 (35.6)
Day 2	97.5 (3.00)	88.5 (16.4)	97.8 (4.29)	98.3 (3.47)	93.2 (10.9)	90.6 (17.3)	89.8 (11.6)
Day 8	93.1 (5.64)	96.6 (5.31)	96.1 (2.95)	97.9 (3.98)	99.8 (0.408)	97.9 (5.03)	97.8 (2.64)
Day 22 predose	—	—	—	—	—	95.2 (5.98)	96.3 (4.38)
Day 29 predose	94.7 (6.18)	86.4 (10.3)	90.1 (9.46)	95.7 (5.74)	93.0 (5.62)	—	—
Day 43 predose	—	—	—	—	—	92.1 (5.15)	95.9 (4.52)
Day 57 predose	90.9 (11.4)	90.9 (14.6)	99.2 (1.08)	90.9 (5.31)	93.2 (4.95)	—	—
Day 85 predose	88.3 (12.1)	96.2 (5.79)	93.8 (4.60)	97.1 (5.02)	97.3 (2.30)	—	96.6 (6.11)
Day 127 predose	89.6 (13.4)	98.5 (2.95)	95.4 (5.37)	94.6 (0.283)	98.1 (3.46)	84.3 (−)	86.5 (10.9)
Day 169 predose	89.1 (10.2)	97.4 (4.50)	90.9 (5.37)	94.0 (2.46)	96.0 (6.87)	92.5 (−)	94.7 (3.11)
Day 211 predose	96.4 (2.31)	86.5 (6.93)	93.1 (2.14)	93.5 (3.54)	100 (0.00)	88.8 (−)	97.8 (−)
Day 253 predose	100 (0.00)	98.8 (1.77)	—	97.3 (−)	91.3 (12.3)	100 (−)	—
**Percentage change from baseline in serum VEGF level (%)**							
Day 1 predose	0.00 (0.00)	0.00 (0.00)	0.00 (0.00)	0.00 (0.00)	0.00 (0.00)	0.00 (0.00)	0.00 (0.00)
Day 1 EOI	—	−95.9 (2.62)	−96.4 (−)	−48.6 (107)	−94.2 (2.67)	162 (628)	−89.1 (9.69)
Day 2	−63.3 (43.6)	−61.1 (27.5)	−75.7 (27.3)	−80.2 (35.0)	—	—	—
Day 8	−35.2 (63.9)	−40.6 (46.9)	−21.9 (85.5)	−2.61 (158)	−70.1 (10.8)	60.8 (324)	−44.1 (37.5)
Day 22 predose	—	—	—	—	—	57.8 (310)	−32.9 (49.8)
Day 22 EOI	—	—	—	—	—	−74.6 (46.5)	−88.5 (9.30)
Day 29 predose	−48.9 (46.3)	−37.0 (56.8)	−33.9 (51.2)	14.6 (200)	−51.6 (18.1)	—	—
Day 29 EOI	−92.9 (−)	−93.5 (3.09)	−82.8 (−)	−86.3 (14.5)	−86.7 (5.36)	—	—
Day 43 predose	−68.6 (−)	−52.3 (53.8)	−36.4 (74.7)	−46.0 (53.0)	−49.6 (17.9)	88.6 (340)	−22.5 (55.1)
Day 43 EOI	−90.7 (−)	−93.9 (3.88)	−85.0 (18.2)	−83.6 (16.9)	−85.1 (6.09)	−72.1 (47.4)	−84.3 (13.4)
Day 85 predose	−66.5 (15.1)	−42.5 (33.5)	−19.3 (110)	4.88 (110)	−61.3 (16.6)	−66.0 (−)	−20.5 (57.7)
Day 85 EOI	−90.1 (2.31)	−83.4 (9.46)	−77.2 (26.9)	−76.9 (16.3)	−88.0 (7.42)	−92.8 (−)	−74.9 (33.5)
Day 127 predose	−47.2 (38.8)	−32.7 (35.5)	−66.7 (10.5)	−51.3 (28.8)	−61.2 (21.2)	−56.4 (−)	51.7 (141)
Day 127 EOI	−85.6 (7.11)	−79.9 (11.1)	−89.9 (3.38)	−85.1 (5.88)	−87.7 (10.1)	−92.3 (−)	−65.5 (40.0)
Day 169 predose	−60.0 (12.7)	−31.8 (44.4)	−73.8 (14.7)	−55.3 (14.9)	−67.6 (14.6)	−57.6 (−)	−40.0 (28.7)
Day 169 EOI	−88.3 (3.38)	−79.7 (11.5)	−92.7 (5.42)	−85.3 (4.68)	−90.3 (6.08)	−93.3 (−)	−87.9 (3.10)
Day 211 predose	−49.4 (32.2)	−60.1 (39.9)	−74.4 (11.4)	−64.1 (6.58)	−44.5 (31.3)	−48.1 (−)	−64.4 (−)
Day 211 EOI	−85.5 (8.53)	−49.0 (39.2)	−92.9 (4.04)	−89.6 (3.76)	−86.2 (5.57)	−89.0 (−)	−91.6 (−)
Day 253 predose	−49.7 (21.4)	−73.5 (31.9)	—	−65.4 (−)	59.1 (118)	−55.5 (−)	—
Day 253 EOI	−87.3 (0.426)	−90.9 (6.487)	—	−92.2 (−)	−84.6 (8.95)	−90.9 (−)	—

*Note:* Data were expressed as mean (SD).

Abbreviations: EOI, end of infusion; PD‐1, programmed death‐1; Q2W, every 2 weeks; Q3W, every 3 weeks; VEGF, vascular endothelial growth factor.

Serum VEGF level decreased after the first dose in all cohorts except the 10 mg/kg Q3W cohort, compared to the baseline (Figure 4B and Table 5). The mean percentage changes from baseline for these cohorts within 1 day after the first dose were up to −63.3%, −95.9%, −96.4%, −80.2%, −94.2%, and − 89.1%, respectively. At Day 8, a gradual increase was observed, and the mean percentage changes from baseline elevated to −35.2%, −40.6%, −21.9%, −2.61%, −70.1%, and − 44.1%, respectively. Before the second dose, the mean percentage changes from baseline were − 48.9%, −37.0%, −33.9%, 14.6%, −51.6%, and − 32.9%, respectively. The trend of initial decrease followed by gradual increase of serum VEGF level were observed after each ivonescimab administration. In addition, these changes in serum VEGF level were not associated with the escalating dose of ivonescimab.

However, serum VEGF level at 10 mg/kg Q3W increased from baseline after the first dose (Figure 4B and Table 5). At the end of the first dose, the mean percentage change from baseline was 162%, and it remained higher than those of other cohorts at the first 3 cycles.

## 4 Discussion

In this study, ivonescimab was investigated for the first time in Chinese patients suffered from advanced solid tumors. The treatment was well‐tolerated across all dose cohorts, with both single and multiple administrations. Only one patient in the 10 mg/kg Q2W cohort experienced possibly ivonescimab‐induced CTCAE Grade 3 immune‐mediated cholangitis after one infusion, which was the only one DLT observed in this study. This patient died of respiratory failure that was not related to ivonescimab after withdrawing from the study. No more cases of immune‐mediated cholangitis were reported in other patients, even at doses up to three times higher. As a result, the MTD of ivonescimab was not reached in this study.

Fifty‐three patients (89.8%) reported at least one TRAE. The most common TRAEs were proteinuria and aspartate aminotransferase increased. No unexpected safety signals were identified, except for known AEs associated with other anti‐PD‐1/PD‐L1/VEGF antibodies. A lower incidence of ≥ Grade 3 TRAEs (23.7%) and serious TRAEs (11.9%) was observed compared with those in the IMbrave150 study (45.3% and 23.1%) [20], in which bevacizumab (an anti‐VEGF agent) plus atezolizumab (a PD‐L1 inhibitor) was used. The incidence of ≥ Grade 3 TRAEs (23.7%) in this study was comparable to that in a phase 2 study of bevacizumab plus nivolumab (a PD‐1 inhibitor) [7]. Of all the TRAEs, no deaths were reported. Additionally, the safety profile remained consistent across ivonescimab doses in the range of 3–30 mg/kg, suggesting the incidence of AEs was not dose‐dependent. The safety profile of ivonescimab in this study among Chinese patients was consistent with the known profile established in the first‐in‐human study (AK112‐101) involving Australian patients [16]. Overall, no apparent safety concerns were indicated for the further development of ivonescimab. The following phase Ib, phase II, and phase III studies of ivonescimab as monotherapy or in combination with chemotherapy in patients with advanced NSCLC [17, 18, 19] have further demonstrated acceptable safety profiles in a larger sample size.

The ivonescimab was rapidly absorbed after a single dose with a median T_max_ of 1.10–3.95 h. Thereafter, ivonescimab was slowly eliminated from the body with a mean *t*_1/2_ of 4.98–7.30 days and a mean CL of 0.328–0.491 L/day. Following multiple dosing, the median *T*_max,ss_ and mean CL_ss_ were 1.00–4.60 h and 0.307–0.414 L/day, respectively, suggesting similar PK characteristics to those after a single dose. No apparent dose‐dependence was found in PK parameters, such as *T*_max_, *t*_1/2_, and CL between 3 and 30 mg/kg. Nevertheless, the *C*_max_ and AUC of ivonescimab increased in a dose‐related manner from 3 to 30 mg/kg. Statistical analysis demonstrated that the slopes (β) for C_max_ and AUC were near 1 and the 90% CI for β included 1, suggesting the relationship between exposure and dose was approximately proportional.

Recently published AK112‐101 study reported PK profile of ivonescimab following single‐dose and multiple‐dose administrations in Australian patients [16]. After the first dosing, the mean *C*_max_ for 3, 10, 20, and 30 mg/kg Q2W cohorts was 77.8, 240, 511, and 672 μg/mL, respectively; the mean AUC_0‐t_ was 409, 1220, 2790, and 3580 day·μg/mL, respectively; and the mean t_1/2_ was 4.53, 6.13, 6.60, and 6.03 days, respectively, which were similar to those reported in the current study in Chinese patients. This suggests that there is no evidence of PK differences between Chinese and non‐Chinese populations for ivonescimab monotherapy.

The mean accumulation ratios of C_max_ and AUC for all dose cohorts ranged between 1.16–1.49 and 1.13–1.68, respectively, indicating mild accumulation of ivonescimab. Furthermore, with administration every 2 or 3 weeks, the mean trough concentration of ivonescimab appeared to be relatively constant following approximately 5 doses, indicating that a steady state was achieved after the fifth dose during treatment.

Treatment‐emergent ADAs were detected in three patients (5.5%), all with neutralizing potential. As expected, no correlation was observed between ADA development and the dosage given. Due to the sample size of this study, the impact of neutralizing ADAs on PK parameters will be explored in the future population PK model.

PD studies explored PD‐1 occupancy and serum VEGF level as an indication of target engagement. The results showed that ivonescimab occupancy on PD‐1 remained high (> 80%) throughout the treatment period and serum VEGF level quickly dropped to near zero after each ivonescimab administration. There were no dose‐dependent changes in PD‐1 occupancy and serum VEGF level. The PD data were consistent with that observed in Australian patients [16]. These findings demonstrate that ivonescimab has a high affinity to PD‐1 and VEGF, making ivonescimab a promising treatment option for advanced solid tumors.

Interestingly, the serum VEGF level gradually came back up after decreasing prior to the next ivonescimab administration. It is well established that bleeding events are major AEs reported in clinical trials of VEGF blocking agents. The disruption of the VEGF signaling pathway, leading to capillary integrity loss in tissues, is a key mechanism behind such bleeding. Bevacizumab, a typical anti‐VEGF agent, is known to maintain constant suppression of VEGF in the serum [21], causing severe hemorrhage‐related events in cancer patients [22]. A meta‐analysis concludes that the incidence of all‐grade hemorrhage in 1979 patients receiving bevacizumab is 30.4% (95% CI, 21.5%–40.9%) [23]. In contrast, ivonescimab has a shorter half‐life compared to the 20‐day half‐life of bevacizumab and allows partial recovery of serum VEGF level at the later part of each treatment cycle, which may help to relieve the disruption of VEGF signaling pathway and potentially reduce the associated toxicities. In our study, ≥ Grade 3 TRAEs specifically related to the VEGF target included hypertension (5.1%), proteinuria (3.4%), and deep vein thrombosis (1.7%). There were no ≥ Grade 3 hemorrhagic‐related TRAEs. While in the bevacizumab label, Grade 3–4 hypertension incidence varied from 5% to 18%, Grade 3–4 proteinuria incidence ranged from 0.7% to 7%, Grade 3–4 venous thromboembolic events incidence ranged from 5% to 11%, and Grade 3–5 hemorrhagic events incidence ranged from 0.4% to 7% in clinical studies. Indeed, our results demonstrate that the incidence of adverse events specifically related to the VEGF target with ivonescimab is significantly lower than that with bevacizumab.

In addition, elevated serum VEGF levels were observed at the 10 mg/kg Q3W cohort during the first 3 cycles, this phenomenon could be attributed to individual variation. One subject in this cohort had a baseline VEGF level below quantification limit (BQL; < 15.6 pg/mL). Normal tissue metabolism requires a certain level of VEGF, thus the feedback regulation increases VEGF level. Consequently, a high VEGF level of 241 pg/mL was manifested after the first dose in this subject. However, the baseline VEGF levels of other 5 subjects in this cohort were greater than 100 pg/mL, and then decreased to BQL after the first dose. As a result, the mean VEGF level of these 6 subjects in the 10 mg/kg Q3W cohort after the first dose showed an upward trend compared to baseline during the first 3 cycles.

Overall, the safety, PK, and PD profiles of ivonescimab in Chinese patients with advanced solid tumors are similar to those observed in patients in Australia in the AK112‐101 study. This finding will bolster our understanding and support the efficacy of ivonescimab. It should be acknowledged that the results from current study have a few limitations. First, the small sample size with a variety of solid tumors could limit the interpretation of the findings. Second, the absence of a control group limits the ability to draw definitive conclusions regarding the safety and efficacy of ivonescimab. Third, this study only included Chinese population, which limits the understanding of the safety, PK, and PD profiles of ivonescimab in solid tumors. Therefore, it is necessary that future clinical studies address these uncertainties and provide a more comprehensive understanding of ivonescimab.

The efficacy data will be published in the future. We believe that these results will provide valuable insights into the PK, and PD profiles of ivonescimab in solid tumors.

## 5 Conclusions

In summary, ivonescimab monotherapy has a good safety profile, demonstrates linear pharmacokinetic characteristics, and sustains high level of receptor occupancy in patients with advanced solid tumors. Ivonescimab represents a new, feasible, promising treatment option for advanced solid tumors.

## Author Contributions

**Fenghua Wang:** investigation (equal), writing – review and editing (equal). **Xiaoli Wei:** investigation (equal), writing – review and editing (equal). **Yulong Zheng:** investigation (equal), writing – review and editing (equal). **Jing Wang:** investigation (equal), writing – review and editing (equal). **Jieer Ying:** investigation (equal), writing – review and editing (equal). **Xiaozhong Chen:** investigation (equal), writing – review and editing (equal). **Suxia Luo:** investigation (equal), writing – review and editing (equal). **Huiyan Luo:** investigation (equal), writing – review and editing (equal). **Xufang Yu:** conceptualization (equal), data curation (equal), formal analysis (equal), funding acquisition (equal), methodology (equal), visualization (equal), writing – original draft (equal), writing – review and editing (equal). **Benchao Chen:** data curation (equal), formal analysis (equal), methodology (equal), visualization (equal), writing – original draft (equal), writing – review and editing (equal). **Lei Ma:** formal analysis (equal), methodology (equal), writing – review and editing (equal). **Ruihua Xu:** supervision (equal), writing – review and editing (equal).

## Ethics Statement

The protocol of this study was reviewed and approved by Ethics Committee of Sun Yat‐sen University Cancer Center (Number A2020‐064‐01), Ethics Committee of The First Affiliated Hospital, Zhejiang University School of Medicine (Number PRO20200206), Ethics Committee of Zhejiang Cancer Hospital (Number IRB‐[2020]932), Ethics Committee of Hunan Cancer Hospital (Number 2020LP00164), and Ethics Committee of Henan Cancer Hospital (Number 2020–231‐001).

## Consent

Each patient signed an informed consent prior to their participation in the study.

## Conflicts of Interest

Xufang Yu, Benchao Chen, and Lei Ma are employees of Akeso Biopharma Inc. The remaining authors declare no conflicts of interest.

## Supporting information


Data S1:

## Data Availability

The data that support the findings of this study are available on a reasonable request from the corresponding author.
