# Supplementary material for: Safety, Pharmacokinetics, and Pharmacodynamics Evaluation of Ivonescimab, a Novel Bispecific Antibody Targeting PD‐1 and VEGF, in Chinese Patients With Advanced Solid Tumors
Source: Cancer Med. 2025 Mar 20;14(6):e70653. doi: 10.1002/cam4.70653 (PMC11925807; doi:10.1002/cam4.70653)
Supplement: Supplementary file 1 — Data S1: [file CAM4-14-e70653-s001.docx]

**Supplementary Table S1** DLT definitions

| **DLTs evaluated to be related to ivonescimab** |
| --- |
| Hepatic and nonhematologic toxicity (treatment-related)   - Grade 4 AST/ALT elevation - AST/ALT ≥ 5 × but ≤ 8 × ULN that does not improve to Grade 2 within 5 days after onset, despite optimal medical management including systemic corticosteroids - AST/ALT > 8 × ULN or TBL > 5 × ULN, regardless of duration or reversibility - Concurrent elevation of AST/ALT > 3 × ULN and TBL > 2 × ULN (i.e., Hy’s Law) with no alternative etiology   Nondermatologic, nonhepatic, and nonhematologic toxicity (treatment-related)  Any ≥ Grade 3 treatment-related nondermatologic, nonhepatic, and nonhematologic toxicity will be considered as a DLT, except for the following specific cases:   - Grade 3 fatigue lasting ≤ 7 days - Grade 3 diarrhea, nausea, or vomiting that has resolved to ≤ Grade 2 within 72 hours after appropriate clinical management - Grade 3 endocrine disorders in asymptomatic subjects with or without systemic corticosteroid therapy and/or hormone replacement therapy - Grade 3 inflammation attributed to local anti-tumor response (e.g., inflammation at metastatic sites or lymph nodes) - Grade 3 or 4 hyperglycemia that has resolved to ≤ Grade 2 within 72 hours after standard hypoglycemic treatment - Isolated Grade 3 amylase or lipase elevation not associated with clinical signs or symptoms or radiographic features suggestive of pancreatitis - Isolated Grade 3 fever not associated with hemodynamic compromise (e.g., hypotension and clinical or laboratory evidence of impaired end-organ perfusion) - Grade 3 infusion-related reactions (first occurrence and without steroid prophylaxis) that relieves within 6 hours after appropriate clinical management - Grade 3 electrolyte alteration without related clinical adverse experiences and reversible to ≤ Grade 1 within 72 hours after onset   Any other ≥ Grade 3 treatment-related nonhematologic toxicity requiring medical intervention, or resulting in hospitalization or prolongation of the hospital stay, or abnormality lasting for > 72 hours will be considered as a DLT.  Dermatologic toxicity (treatment-related)   - Grade 3 rash with no improvement (i.e., resolution to ≤ Grade 1) after 2 weeks of infusion - Grade 4 rash of any duration   Hematologic toxicity (treatment-related)   - Grade 4 neutropenia lasting ≥ 7 days or requiring treatment with granulocyte-colony stimulating factor - Grade 4 thrombocytopenia - Grade 3 thrombocytopenia with bleeding - ≥ Grade 3 febrile neutropenia of any duration - ≥ Grade 3 hemolysis (i.e., requiring transfusion or medical intervention such as steroids) |

DLT, dose-limiting toxicity; ULN, upper limit of normal; AST, aspartate aminotransferase; ALT, alanine aminotransferase; TBL, total bilirubin.

**Supplementary Table S2** Full inclusion/exclusion criteria

| **Inclusion criteria** |
| --- |
| Subjects must meet all of the following criteria:   1. A signed written informed consent by the subject/legal representative must be obtained prior to performing any protocol-related procedures including screening evaluations. 2. Age ≥ 18 and ≤ 75 years at the time of signing the written informed consent. 3. Subjects with histologically or cytologically confirmed advanced or metastatic solid tumors who have failed at least one prior standard therapy in the advanced or metastatic stage (subjects who experience disease progression or recurrence within 6 months after the end of neo-adjuvant or adjuvant therapy will be considered to have failed a standard therapy), or have no effective standard therapy available, or are unable to tolerate or refuse to receive standard therapy. (For subjects in the dose-expansion phase, requirements for prior treatment can be found in other inclusion criteria.) 4. Dose-escalation phase: 5. Dose-escalation cohorts: All subjects with solid tumors. 6. Cohorts expanded for additional enrollment: Subjects with advanced or metastatic non-small cell lung cancer (NSCLC), small cell lung cancer (SCLC), recurrent/refractory epithelial ovarian cancer (including primary peritoneal and fallopian tube cancers), persistent/recurrent or metastatic cervical cancer, metastatic colorectal cancer, renal cell carcinoma (RCC), hepatocellular carcinoma (HCC), gastric cancer, esophageal cancer, microsatellite instability-high (MSI-H) or mismatch repair deficient (dMMR) cancer, urothelial cancer, metastatic triple-negative breast cancer (TNBC), mesothelioma, or endometrial cancer. Other tumor types may be considered after discussion with the sponsor. 7. In the dose-expansion phase, all subjects must have received up to a maximum of third-line systemic therapy for advanced or metastatic diseases (with the possibility of considering subjects who have received systemic therapy beyond the third line after discussion and agreement with the sponsor): 8. Recurrent/refractory epithelial ovarian cancer (including primary peritoneal and fallopian tube cancers):  - Pathologically confirmed ovarian epithelial malignancy, fallopian tube epithelial malignancy, and primary peritoneal cancer. - Subjects have received ≥ first-line platinum-based chemotherapy, and meet any of the following criteria: 1) Platinum-resistant or refractory patients who experience disease progression or recurrence during or within 6 months after the end of platinum-based chemotherapy; 2) Platinum-sensitive patients (i.e., disease progression or recurrence ≥ 6 months after the end of platinum-based chemotherapy) who experience disease progression or recurrence after receiving at least 2 different platinum-based chemotherapies (including the initial platinum-based chemotherapy). Note: Evidence of objective radiological or clinical disease progression (e.g., cytological report of new ascites or pleural effusion) should be provided. Only an increase in tumor biomarkers cannot be considered as disease progression or recurrence in this study.  1. NSCLC:  - Locally advanced or metastatic NSCLC that cannot be completely surgically resected, confirmed histologically or cytologically according to the 8th edition of the TNM staging classification for lung cancer, and does not contain any components of SCLC. - For squamous NSCLC, subjects with central and cavitary squamous NSCLC who have a high risk of bleeding judged by the investigator cannot be enrolled; if subjects’ EGFR and ALK genotypes are unknown before enrollment, testing is not required. - For non-squamous NSCLC, subjects’ EGFR and ALK must be wild-type (if not previously tested, testing is required before enrollment).  1. HCC:  - Pathologically confirmed HCC without components such as fibrolamellar hepatocellular carcinoma, sarcomatoid hepatocellular carcinoma, and cholangiocarcinoma; - Barcelona Clinic Liver Cancer (BCLC) stage C, and stage B that is not suitable for curative surgery and/or local treatment, or disease progression after surgery and/or local treatment; - Subjects have received at most one type of anti-angiogenic therapy or PD-1/L1 inhibitor for HCC and failed or were intolerant; - Child-Pugh A liver function; - No esophageal or gastric variceal bleeding due to portal hypertension within 6 months prior to the first dose of investigational product; - Subjects have undergone esophagogastroduodenoscopy within 6 months prior to the first dose of investigational product, and if severe (G3) varices are found, participation in this study is not allowed.  1. Metastatic colorectal cancer:  - Histologically confirmed metastatic colon or rectal adenocarcinoma, with metastatic/recurrent lesions that cannot be cured by surgery.  1. Gastric and gastroesophageal junction adenocarcinoma:  - Histologically confirmed unresectable locally advanced, recurrent, or metastatic gastric and gastroesophageal junction adenocarcinoma; - Human epidermal growth factor 2 (HER2)-positive gastric or gastroesophageal junction adenocarcinoma must be subjects who have received HER2 inhibitor and failed.  1. RCC:  - Histologically confirmed unresectable or recurrent/metastatic RCC containing clear cell components, with or without sarcomatoid components (after obtaining permission from the sponsor, enrollment of non-clear cell RCC, such as papillary renal cell carcinoma and chromophobe renal cell carcinoma, is allowed); - Subjects have received at most one type of anti-angiogenic therapy or PD-1/L1 inhibitor for RCC and failed or were intolerant.  1. TNBC:  - Histologically confirmed locally advanced or metastatic TNBC (i.e., HER2, estrogen receptor [ER], and progesterone receptor [PR] negative), not suitable for curative surgical resection or local treatment, or disease progression after surgical resection or local treatment; - Subjects have not received prior systemic anti-tumor therapy; - Subjects who have received chemotherapy for early breast cancer (neo-adjuvant or adjuvant therapy) but have relapsed or metastasized ≥ 12 months after completing chemotherapy before the first dose of investigational product can participate in this study.  1. Subject must have at least one measurable lesion according to RECIST v1.1. A previously irradiated lesion can be considered a target lesion if it is well defined, measurable per RECIST v1.1, and there is objective evidence of significant progression since radiotherapy. 2. Eastern Cooperative Oncology Group (ECOG) performance status of 0 or 1. 3. Life expectancy ≥ 12 weeks. 4. If archival tumor tissue samples are available (formalin-fixed paraffin-embedded [FFPE] tissue blocks or at least 10 unstained FFPE tissue slides are mandatory for the expanded cohorts in phase I and the dose-expansion cohorts in phase II, but may not be required for the dose-escalation cohorts in phase I after communication with the sponsor’s medical monitor), they must be provided for tumor-related biomarker analyses. In cases where archival material is not available or not suitable for use (e.g., in subjects recently diagnosed or diagnosed by fine needle aspiration), subjects should consent and undergo a new tumor biopsy (at the investigator’s discretion, with acceptable risk). 5. In order to explore the correlation between different immune cell subtypes infiltrating the tumor and efficacy, subjects need to provide an additional 10 unstained FFPE tumor tissue slides. If unable to provide, after approval from the medical monitor, this requirement may be waived without affecting the subject’s participation in the study. 6. Adequate bone marrow reserves and organ function as determined by: 7. Hematological (no blood transfusion within 14 days prior to screening, no use of granulocyte-colony stimulating factor, and no medication to correct):  - Absolute neutrophil count (ANC) ≥ 1.5 × 10^9^/L (1500/mm^3^); - Platelet count ≥ 100 × 10^9^/L (100,000/mm^3^); for subjects with HCC, platelet count ≥ 75 × 10^9^/L (75,000/mm^3^); - Hemoglobin ≥ 9.0 g/dL within the first 2 weeks prior to the first dose of investigational product.  1. Renal:  - Creatinine clearance (CrCl) ≥ 50 mL/min (Cockcroft-Gault formula will be used to calculate CrCl; CrCL (mL/min) = {(140 - age) × weight (kg) × F}/ (SCr (mg/dL) × 72), where F = 1 for males, F = 0.85 for females, and SCr = serum creatinine); - Urine dipstick for proteinuria < 2+ (i.e., 0, trace, or 1+). Note: All patients with ≥ 2+ protein on dipstick urinalysis must undergo a 24-hour urine collection and must demonstrate ≤ 1 g of protein in 24 hours.  1. Hepatic:  - Total bilirubin (TBL) ≤ 1.5 × upper limit of normal (ULN); for subjects with HCC, liver metastasis, or documented/suspected Gilbert’s disease, TBL ≤ 3 × ULN; - AST and ALT ≤ 2.5 × ULN; for subjects with liver metastasis or HCC, AST and ALT ≤ 5 × ULN without elevated bilirubin); - Serum albumin (ALB) ≥ 28 g/L.  1. Coagulation:  - International normalized ratio (INR) and activated partial thromboplastin time (APTT) ≤ 1.5 × ULN (unless the subject is receiving anticoagulant therapy and coagulation parameters (PT/INR and APTT) are within the expected range for anticoagulant therapy at screening).  1. Females of childbearing potential must undergo a urine or serum pregnancy test within 3 days prior to the first dose of investigational product (if the urine pregnancy test result cannot be confirmed as negative, a serum pregnancy test should be performed, with the serum pregnancy result being definitive), and the result must be negative. Females of childbearing potential who are sexually active with non-sterilized male partners, and non-sterilized males who are sexually active with female partners of childbearing potential must agree to use effective contraception methods during the study and for 3 months after the last dose of investigational product, such as abstinence, dual barrier contraceptive methods, contraceptive condoms, oral or injectable contraceptives, intrauterine devices, etc. 2. Females of childbearing potential are defined as those who are not surgically sterile (i.e., bilateral tubal ligation, bilateral oophorectomy, or complete hysterectomy) or postmenopausal (menopause is defined as the absence of menstruation for at least 12 consecutive months without an alternative medical cause, and serum estrogen and follicle-stimulating hormone levels are within the laboratory reference range for postmenopausal women). 3. A highly effective method of contraception* is defined as one that results in a low failure rate (i.e., < 1% per year) when used consistently and correctly. Not all contraception methods are highly effective. Female subjects must use a hormonal method (e.g., the contraceptive pill) in addition to a barrier method (e.g., male condom plus spermicide) alone, to ensure pregnancy does not occur. Note: *Highly effective methods of contraception are as follows: 1) Barrier method: male condom plus spermicide, copper T intrauterine device, and levonorgestrel-releasing intrauterine device (e.g., Mirena); 2) Hormonal method: implants, hormone shot or injection, combined pill, minipill, and patch. 4. Should a woman become pregnant or suspect she is pregnant while she or her partner is participating in this study, she must inform her treating physician immediately. |
| **Exclusion criteria** |
| Subjects who meet any of the following criteria will be ineligible for participation in the study:   1. History of severe hypersensitivity reactions to other monoclonal antibodies or bispecific antibodies. 2. Active malignancy within the past 5 years, except for the tumor for which a subject is enrolled in the study, and locally curable cancers that have been apparently cured such as basal or squamous cell skin cancer, superficial bladder cancer, or carcinoma in situ of the cervix or breast. 3. Concurrent enrollment in another clinical study, unless it is an observational (non-interventional) clinical study or the follow-up period of an interventional study. 4. Receipt of last systemic anti-tumor therapy (such as chemotherapy, immunotherapy, biologic therapy, etc.) within 3 weeks, or treatment with small-molecule tyrosine kinase inhibitor within 2 weeks, or palliative local therapy for non-target lesions within 2 weeks, or non-specific immunomodulatory therapy (interleukins, interferons, thymosin, etc., excluding IL-11) within 2 weeks, or herbal or traditional Chinese medicine with anti-tumor indications within 1 week prior to the first dose of investigational product. For HCC, receipt of local-regional therapy of the liver (such as transarterial chemoembolization, transcatheter embolization, hepatic artery infusion, radiotherapy, radioembolization, or ablation) within 3 weeks prior to the first dose of investigational product. 5. Presence of brainstem or leptomeningeal metastases, or spinal cord metastasis or compression. 6. Active central nervous system metastases; subjects with previously treated brain metastases (e.g., surgery or radiotherapy), who are clinically stable for at least 2 weeks (counting from the start of taking the investigational product) and discontinued corticosteroids for 7 days prior to the first dose of investigational product, are allowed to participate; subjects with untreated and asymptomatic brain metastases (i.e., no neurological symptoms, no need for corticosteroids, no brain metastases with a long axis > 1.5 cm, and no significant peritumoral edema) are eligible for inclusion. 7. Pleural effusion, pericardial effusion, or ascites with clinical symptoms or requiring repeated drainage. 8. Imaging at screening shows that tumor surrounds important blood vessels or has obvious necrosis or cavities, and the investigator determines that participation in the study would cause a risk of bleeding during the study period. 9. History or concurrent gastrointestinal perforation, surgery, wound healing complications, and bleeding: 10. History of abdominal or tracheoesophageal fistula, gastrointestinal perforation, abdominal fistula, or intra-abdominal abscess within 6 months prior to the first dose of investigational product; 11. Presence of gastrointestinal obstruction or requirement for routine parenteral hydration, parenteral nutrition, or tube feeding; 12. Severe non-healing wounds, active ulcers, or untreated bone fractures; 13. Presence of intra-abdominal free air not explained by paracentesis or recent surgery; 14. History of hemoptysis (≥ 1 teaspoon of bright red blood or small clots per episode; subjects with blood-tinged sputum are included) or acute gastrointestinal bleeding within 1 month prior to the first dose of investigational product; 15. Bleeding diathesis or coagulation disorders (in the absence of therapeutic anticoagulation). 16. Subjects with clinically significant cardiovascular diseases: 17. Poorly controlled hypertension (defined as systolic blood pressure [SBP] > 150 mmHg and/or diastolic blood pressure [DBP] > 100 mmHg). Note: Anti-hypertensive therapy to achieve these parameters is allowable. 18. History of hypertensive crisis or hypertensive encephalopathy; 19. History of myocarditis or cardiomyopathy; 20. Major vascular disease (e.g., aortic aneurysm requiring surgery, any arterial thrombosis, or NCI CTCAE v5.0 Grade 3 or higher venous thromboembolism) within 6 months prior to the first dose of investigational product; 21. Myocardial infarction or unstable angina within 6 months prior to the first dose of investigational product; 22. New York Heart Association (NYHA) grade II or higher congestive heart failure; 23. Severe cardiac arrhythmia requiring medication (asymptomatic atrial fibrillation with controlled ventricular rate can be included); 24. History of cerebrovascular disease within 6 months prior to the first dose of investigational product; 25. Left ventricular ejection fractions (LVEF) < 50%. 26. Concurrent uncontrolled comorbid conditions, such as decompensated cirrhosis, nephrotic syndrome, uncontrolled metabolic disorders, severe active peptic ulcer disease or gastritis, or psychiatric/social conditions that would limit the subject’s compliance with study procedures or impair the subject’s ability to provide written informed consent. 27. Subjects who have received prior anti-PD-1, anti-PD-L1, anti-CTLA-4, or any other immunotherapy or immune-oncology (IO) agents: 28. Subjects have received anti-PD-1, anti-PD-L1, anti-CTLA-4, or any other immunotherapy or IO agents within 28 days prior to the first dose of investigational product. 29. Subjects have experienced immune-related adverse events (irAEs) leading to permanent discontinuation of prior immunotherapy. Subjects have experienced NCI CTCAE v5.0 ≥ Grade 3 irAEs or neurologic or ocular AEs of any grade while receiving prior immunotherapy. 30. All AEs while receiving prior immunotherapy have not completely resolved or only resolved to Grade 1 prior to screening for this study. Subjects with ≥ Grade 2 endocrine AEs are permitted to participate if they are stably maintained on appropriate replacement therapy and are asymptomatic. 31. Subjects have required the use of high-dose glucocorticoid (≥ 40 mg/day prednisone or equivalent) or other immunosuppressive medications for the management of AEs, or have experienced a recurrence of AEs requiring re-initiation of glucocorticoid while receiving prior immunotherapy. 32. Subjects who have received prior anti-PD-1, anti-PD-L1, anti-CTLA-4, or any other antibodies or drugs targeting T cell co-stimulation or checkpoint pathways (such as ICOS), or various agonists (such as CD40, CD137, GITR, OX40, etc.) are enrolled in the dose-expansion phase (phase II) with sponsor approval. 33. Subjects with a condition requiring systemic treatment with either glucocorticoid (> 10 mg/day prednisone or equivalent) or other immunosuppressive medications within 7 days prior to the first dose of investigational product. The following are exceptions to this criterion: 34. Intranasal, inhaled, topical, or local glucocorticoid injections (e.g., intra-articular injection); 35. Systemic glucocorticoid at physiologic doses not exceeding 10 mg/day prednisone or equivalent; 36. Glucocorticoid as premedication for hypersensitivity reactions (e.g., premedication for CT or MRI scan). 37. Current or recent (within 10 days prior to the first dose of investigational product) use of aspirin (> 325 mg/day) or treatment with ticlopidine, clopidogrel, and cilostazol. 38. Current use of full-dose oral or parenteral anticoagulants or thrombolytic medications for therapeutic purposes that has not been stable for > 2 weeks prior to the first dose of investigational product: 39. The use of full-dose oral or parenteral anticoagulants is allowed as long as the INR or APTT is within therapeutic limits (according to the medical standard of the enrolling institution) and the patient has been on a stable dose of anticoagulants for at least 2 weeks prior to the first dose of investigational product. 40. Prophylactic anticoagulation for the patency of venous access devices is allowed. 41. Prophylactic use of low-molecular-weight heparin (i.e., 40 mg/day enoxaparin) is allowed. 42. Active autoimmune disease requiring systemic therapy (e.g., disease-modifying drugs, corticosteroids, or immunosuppressive medications) in the past 2 years; replacement therapy (e.g., thyroid hormone, insulin, or physiological corticosteroid replacement therapy for adrenal or pituitary insufficiency) is not considered a systemic therapy. 43. Active or prior documented inflammatory bowel disease (e.g., Crohn’s disease, or ulcerative colitis). 44. History of immunodeficiency; positive HIV antibody test; currently on long-term systemic corticosteroids or other immunosuppressive therapy. 45. History of organ or hematopoietic stem cell transplantation requiring the use of immunosuppressive therapy. 46. Known allergy or reaction to any component of the investigational product. 47. History of non-infectious pneumonitis or interstitial lung disease requiring systemic glucocorticoid therapy, or currently present with non-infectious pneumonia. 48. Unresolved toxicities from prior anti-tumor therapy, defined as having not resolved to NCI CTCAE v5.0 Grade 0 or 1 (except for alopecia), or to levels dictated in the inclusion/exclusion criteria. Subjects with irreversible toxicity that is not reasonably expected to be exacerbated by the investigational product may be included (e.g., hearing loss) after consultation with the medical monitor. Subjects with Grade 2 neuropathy will be evaluated on a case-by-case basis after consultation with the medical monitor. 49. Major surgical procedure (as defined by the investigator) within 30 days prior to the first dose of investigational product or still recovering from prior surgery. Core biopsy or other minor surgical procedure, excluding placement of a vascular access device, within 7 days prior to the first dose of investigational product. 50. History of psychiatric illness, substance abuse, alcohol abuse, or drug addiction. 51. Females who are pregnant or lactating. 52. Known active pulmonary tuberculosis; known active syphilis infection. 53. Subjects with untreated active hepatitis B (HBsAg positive, and HBV-DNA > 1000 copies/mL (200 IU/mL) or higher than the lower limit of quantitation, whichever is higher). Note: Subjects with hepatitis B are eligible if they are treated with antiviral therapy during the study period. Subjects with active hepatitis C (HCV antibody positive, and HCV-RNA higher than the lower limit of quantitation). Note: For HCC patients with HBV infection, those with HBV-DNA < 500 IU/mL obtained within 28 days prior to the first dose of investigational product, have received at least 14 days of anti-HBV treatment (according to local standard of care, e.g., entecavir) before enrollment, and are willing to continue treatment during the study period, are allowed to enroll; for HCC patients with HCV infection, those with stable conditions judged by the investigator, and are willing to continue antiviral treatment during the study period, are allowed to enroll. 54. For HCC subjects: tumor volume > 50% of liver volume; portal vein tumor thrombus (Vp4, tumor thrombus involving the main portal vein, or tumor mainly involving the main branch of the portal vein on the contralateral liver lobe or the main branch of the portal vein on both sides), inferior vena cava tumor thrombus; presence of NCI CTCAE v5.0 ≥ Grade 2 hepatic encephalopathy and/or history of hepatic encephalopathy. 55. Receipt of systemic antiviral therapy within 2 weeks prior to the first dose of investigational product (excluding antiviral therapy for hepatitis B or hepatitis C). 56. Receipt of live attenuated vaccination within 30 days prior to the first dose of investigational product. 57. Any condition that, in the opinion of the investigator, would interfere with evaluation of the investigational product or interpretation of subject safety or study results. |

NSCLC, non-small cell lung cancer; SCLC, small cell lung cancer; RCC, renal cell carcinoma; HCC, hepatocellular carcinoma; MSI-H, microsatellite instability-high; dMMR, mismatch repair deficient; TNBC, triple-negative breast cancer; TNM, tumor node metastasis; EGFR, epidermal growth factor receptor; ALK, anaplastic lymphoma kinase; BCLC, Barcelona Clinic Liver Cancer; PD-1, programmed death-1; PD-L1, programmed death-ligand 1; CTLA-4, cytotoxic T-lymphocyte-associated protein 4; HER2, human epidermal growth factor receptor 2; ER, estrogen receptor; PR, progesterone receptor; RECIST, Response Evaluation Criteria in Solid Tumors; ECOG, Eastern Cooperative Oncology Group; FFPE, formalin-fixed paraffin-embedded; ANC, absolute neutrophil count; CrCl, creatinine clearance; SCr, serum creatinine; TBL, total bilirubin; ULN, upper limit of normal; AST, aspartate aminotransferase; ALT, alanine aminotransferase; ALB, albumin; INR, international normalized ratio; APTT, activated partial thromboplastin time; PT, prothrombin time; IL-11, interleukin 11; SBP, systolic blood pressure; DBP, diastolic blood pressure; NCI CTCAE, National Cancer Institute Common Terminology Criteria for Adverse Events; NYHA, New York Heart Association; LVEF, left ventricular ejection fractions; IO, immune-oncology; irAE, immune-related adverse event; AE, adverse event; ICOS, inducible co-stimulator; GITR, glucocorticoid-induced tumor necrosis factor receptor; CT, computed tomography; MRI, magnetic resonance imaging; HBsAg, hepatitis B surface antigen; HBV, hepatitis B virus; HCV, hepatitis C virus.

**Supplementary Table S3** Pharmacokinetic sampling timeline^a^

| **Q2W cohorts^b^** | | | | |
| --- | --- | --- | --- | --- |
| **Cycle** | **Drug administration** | **Day** | **Time relative to drug administration** | **Time window** |
| C1 | 1^st^ dose | D1 | Before the dose | within 30 min |
|  |  |  | End of the dose | + 5 min |
|  |  |  | 3 h after the dose | ± 15 min |
|  |  | D2 | 24 h after the dose | ± 3 h |
|  |  | D3 | 48 h after the dose | ± 12 h |
|  |  | D8 | 168 h after the dose | ± 1 d |
|  | 2^nd^ dose | D15 | Before the dose | within 30 min |
|  |  |  | End of the dose | + 5 min |
| C2 | 3^rd^ dose | D1 | Before the dose | within 30 min |
|  |  |  | End of the dose | + 5 min |
|  | 4^th^ dose | D15 | Before the dose | within 30 min |
|  |  |  | End of the dose | + 5 min |
| C3 | 5^th^ dose | D1 | Before the dose | within 30 min |
|  |  |  | End of the dose | + 5 min |
|  |  |  | 3 h after the dose | ± 15 min |
|  |  | D2 | 24 h after the dose | ± 3 h |
|  |  | D3 | 48 h after the dose | ± 12 h |
|  |  | D8 | 168 h after the dose | ± 1 d |
|  | 6^th^ dose | D15 | Before the dose | within 30 min |
|  |  |  | End of the dose | + 5 min |
| C4 | 7^th^ dose | D1 | Before the dose | within 30 min |
|  |  |  | End of the dose | + 5 min |
| C5 | 9^th^ dose | D1 | Before the dose | within 30 min |
|  |  |  | End of the dose | + 5 min |
| C6 | 11^th^ dose | D1 | Before the dose | within 30 min |
|  |  |  | End of the dose | + 5 min |
| C8 and beyond  (every 8 weeks) | 15^th^ dose and beyond  (every 8 weeks) | D1 | Before the dose | within 30 min |
| End of treatment | | 30 d after the last dose | | ± 3 d |
|  |  | 90 d after the last dose | | ± 7 d |
| **Q3W cohorts^c^** | | | | |
| **Cycle** | **Drug administration** | **Day** | **Time relative to drug administration** | **Time window** |
| C1 | 1^st^ dose | D1 | Before the dose | within 30 min |
|  |  |  | End of the dose | + 5 min |
|  |  |  | 3 h after the dose | ± 15 min |
|  |  | D2 | 24 h after the dose | ± 3 h |
|  |  | D3 | 48 h after the dose | ± 12 h |
|  |  | D8 | 168 h after the dose | ± 1 d |
|  |  | D15 | 336 h after the dose | ± 1 d |
| C2 | 2^nd^ dose | D1 | Before the dose | within 30 min |
|  |  |  | End of the dose | + 5 min |
|  |  | D15 | 336 h after the dose | ± 1 d |
| C3 | 3^rd^ dose | D1 | Before the dose | within 30 min |
|  |  |  | End of the dose | + 5 min |
|  |  |  | 3 h after the dose | ± 15 min |
|  |  | D2 | 24 h after the dose | ± 3 h |
|  |  | D3 | 48 h after the dose | ± 12 h |
|  |  | D8 | 168 h after the dose | ± 1 d |
|  |  | D15 | 336 h after the dose | ± 1 d |
| C4 | 4^th^ dose | D1 | Before the dose | within 30 min |
|  |  |  | End of the dose | + 5 min |
| C5 | 5^th^ dose | D1 | Before the dose | within 30 min |
|  |  |  | End of the dose | + 5 min |
| C6 | 6^th^ dose | D1 | Before the dose | within 30 min |
|  |  |  | End of the dose | + 5 min |
| C8 and beyond  (every 6 weeks) | 8^th^ dose and beyond  (every 6 weeks) | D1 | Before the dose | within 30 min |
| End of treatment | | 30 d after the last dose | | ± 3 d |
|  |  | 90 d after the last dose | | ± 7 d |

^a^Since the dose-expansion phase (phase II) was not undertaken owing to a decision that unrelated to patient safety issues made by the sponsor, its planned pharmacokinetic sampling timeline was not shown.

^b^Ivonescimab was administered every 2 weeks, and every 4 weeks was defined as a treatment cycle.

^c^Ivonescimab was administered every 3 weeks, and every 3 weeks was defined as a treatment cycle.

**Supplementary Table S4** Pharmacodynamic sampling timeline^a^

| **Sample collection for receptor occupancy: Q2W cohorts^b^** | | | | |
| --- | --- | --- | --- | --- |
| **Cycle** | **Drug administration** | **Day** | **Time relative to drug administration** | **Time window** |
| C1 | 1^st^ dose | D1 | Before the dose | within 30 min |
|  |  | D2 | 24 h after the dose | ± 3 h |
|  |  | D8 | 168 h after the dose | ± 1 d |
| C2 | 3^rd^ dose | D1 | Before the dose | within 30 min |
| C3 | 5^th^ dose | D1 | Before the dose | within 30 min |
| C4 | 7^th^ dose | D1 | Before the dose | within 30 min |
| C5 | 10^th^ dose | D1 | Before the dose | within 30 min |
| C7 | 13^th^ dose | D1 | Before the dose | within 30 min |
| C8 | 16^th^ dose | D1 | Before the dose | within 30 min |
| C10 | 19^th^ dose | D1 | Before the dose | within 30 min |
| End of treatment | | 30 d after the last dose | | ± 3 d |
| **Samples collection for receptor occupancy: Q3W cohorts^c^** | | | | |
| **Cycle** | **Drug administration** | **Day** | **Time relative to drug administration** | **Time window** |
| C1 | 1^st^ dose | D1 | Before the dose | within 30 min |
|  |  | D2 | 24 h after the dose | ± 3 h |
|  |  | D8 | 168 h after the dose | ± 1 d |
| C2 | 2^nd^ dose | D1 | Before the dose | within 30 min |
| C3 | 3^rd^ dose | D1 | Before the dose | within 30 min |
| C5 | 5^th^ dose | D1 | Before the dose | within 30 min |
| C7 | 7^th^ dose | D1 | Before the dose | within 30 min |
| C9 | 9^th^ dose | D1 | Before the dose | within 30 min |
| C11 | 11^th^ dose | D1 | Before the dose | within 30 min |
| C13 | 13^th^ dose | D1 | Before the dose | within 30 min |
| End of treatment | | 30 d after the last dose | | ± 3 d |
| **Samples collection for VEGF: Q2W cohorts^b^** | | | | |
| **Cycle** | **Drug administration** | **Day** | **Time relative to drug administration** | **Time window** |
| C1 | 1^st^ dose | D1 | Before the dose | within 30 min |
|  |  |  | End of the dose | + 5 min |
|  |  | D8 | 168 h after the dose | ± 1 d |
| C2 | 3^rd^ dose | D1 | Before the dose | within 30 min |
|  |  |  | End of the dose | + 5 min |
|  | 4^th^ dose | D1 | Before the dose | within 30 min |
|  |  |  | End of the dose | + 5 min |
| C4 | 7^th^ dose | D1 | Before the dose | within 30 min |
|  |  |  | End of the dose | + 5 min |
| C5 | 10^th^ dose | D1 | Before the dose | within 30 min |
|  |  |  | End of the dose | + 5 min |
| C7 | 13^th^ dose | D1 | Before the dose | within 30 min |
|  |  |  | End of the dose | + 5 min |
| C8 | 16^th^ dose | D1 | Before the dose | within 30 min |
|  |  |  | End of the dose | + 5 min |
| C10 | 19^th^ dose | D1 | Before the dose | within 30 min |
|  |  |  | End of the dose | + 5 min |
| End of treatment | | 30 d after the last dose | | ± 3 d |
| **Samples collection for VEGF: Q3W cohorts^b^** | | | | |
| **Cycle** | **Drug administration** | **Day** | **Time relative to drug administration** | **Time window** |
| C1 | 1^st^ dose | D1 | Before the dose | within 30 min |
|  |  |  | End of the dose | + 5 min |
|  |  | D8 | 168 h after the dose | ± 1 d |
| C2 | 2^nd^ dose | D1 | Before the dose | within 30 min |
|  |  |  | End of the dose | + 5 min |
| C3 | 3^rd^ dose | D1 | Before the dose | within 30 min |
|  |  |  | End of the dose | + 5 min |
| C5 | 5^th^ dose | D1 | Before the dose | within 30 min |
|  |  |  | End of the dose | + 5 min |
| C7 | 7^th^ dose | D1 | Before the dose | within 30 min |
|  |  |  | End of the dose | + 5 min |
| C9 | 9^th^ dose | D1 | Before the dose | within 30 min |
|  |  |  | End of the dose | + 5 min |
| C11 | 11^th^ dose | D1 | Before the dose | within 30 min |
|  |  |  | End of the dose | + 5 min |
| C13 | 13^th^ dose | D1 | Before the dose | within 30 min |
|  |  |  | End of the dose | + 5 min |
| End of treatment | | 30 d after the last dose | | ± 3 d |

^a^Since the dose-expansion phase (phase II) was not undertaken owing to a decision that unrelated to patient safety issues made by the sponsor, its planned pharmacodynamic sampling timeline was not shown.

^b^Ivonescimab was administered every 2 weeks, and every 4 weeks was defined as a treatment cycle.

^c^Ivonescimab was administered every 3 weeks, and every 3 weeks was defined as a treatment cycle.

**Supplementary Table S5** Definitions of pharmacokinetic parameters

| **Pharmacokinetic parameters after a single dose** | |
| --- | --- |
| T_max_ | Time to reach the maximum observed concentration |
| C_max_ | Maximum observed concentration |
| AUC_0-t_ | Area under the concentration-time curve from 0 h to the time of the last measurable concentration, calculated using trapezoidal summation method |
| AUC_0-τ_ | Area under the concentration-time curve during a dosing interval, calculated using trapezoidal summation method |
| AUC_0-∞_ | Area under the concentration-time curve from 0 h to infinity: AUC_0-∞_ = AUC_0-t_ + C_t_ / λ_z_, where C_t_ is the last measurable concentration, and λ_z_ is the terminal disposition rate constant |
| AUC__%Extrap_ | Percentage of AUC_0-t_ that is due to extrapolation from the time of the last measurable concentration to infinity: AUC__%Extrap_ = [(AUC_0-∞_ - AUC_0-t_) / AUC_0-∞_] × 100% |
| λ_z_ | Terminal disposition rate constant, estimated by the semi-logarithmic linear regression of the elimination phase concentration versus time |
| t_1/2_ | Terminal elimination half-life: t_1/2_ = ln2 / λ_z_ |
| V_z_ | Volume of distribution based on the terminal phase for intravenous administration: V_z_ = CL / λ_z_ |
| CL | Total clearance of the drug for intravenous administration: CL = Dose / AUC_0-∞_ |
| MRT_0-t_ | Mean residence time from 0 h to the time of the last measurable concentration: MRT_0-t_ = AUMC_0-t_ / AUC_0-t_ - T_inf_ / 2, where AUMC_0-t_ is the area under the moment curve from 0 h to the time of the last measurable concentration, and T_inf_ is the length of infusion |
| MRT_0-∞_ | Mean residence time from 0 h to infinity: MRT_0-∞_ = AUMC_0-∞_ / AUC_0-∞_, where AUMC_0-∞_ is the area under the moment curve from 0 h to infinity |
| **Pharmacokinetic parameters after multiple doses** | |
| T_max,ss_ | Time to reach the maximum observed concentration at steady state |
| C_min,ss_ | Minimum observed concentration at steady state |
| C_max,ss_ | Maximum observed concentration at steady state |
| C_avg_ | Average concentration: C_avg_ = AUC_0-τ,ss_ / τ, where τ is the dosing interval |
| Swing | Swing = (C_max,ss_ - C_min,ss_) / C_min,ss_ |
| Fluctuation% | Fluctuation% = 100 × [( C_max,ss_ - C_min,ss_) / C_avg_] |
| AUC_0-t,ss_ | Area under the concentration-time curve from 0 h to the time of the last measurable concentration at steady state, calculated using trapezoidal summation method |
| AUC_0-τ,ss_ | Area under the concentration-time curve during a dosing interval, calculated using trapezoidal summation method |
| AUC_0-∞,ss_ | Area under the concentration-time curve from 0 h to infinity at steady state: AUC_0-∞,ss_ = AUC_0-t,ss_ + C_t,ss_ / λ_z,ss_, where C_t,ss_ is the last measurable concentration at steady state, and λ_z,ss_ is the terminal disposition rate constant at steady state |
| AUC__%Extrap,ss_ | Percentage of AUC_0-t,ss_ that is due to extrapolation from the time of the last measurable concentration to infinity at steady state: AUC__%Extrap,ss_ = [(AUC_0-∞,ss_ - AUC_0-t,ss_) / AUC_0-∞,ss_] × 100% |
| λ_z,ss_ | Terminal disposition rate constant at steady state, estimated by the semi-logarithmic linear regression of the elimination phase concentration versus time |
| t_1/2,ss_ | Terminal elimination half-life at steady state: t_1/2,ss_ = ln2 / λ_z,ss_ |
| V_ss_ | Volume of distribution based on the terminal phase at steady state for intravenous administration: V_ss_ = CL_ss_ × MRT_0-∞,ss_ |
| CL_ss_ | Total clearance of the drug at steady state for intravenous administration: CL_ss_ = Dose / AUC_0-τ,ss_ |
| MRT_0-∞,ss_ | Mean residence time from 0 h to infinity at steady state: MRT_0-∞,ss_ = AUMC_0-τ,ss_ + t × (AUC_0-∞,ss_ - AUC_0-t,ss_) / AUC_0-t,ss_, where AUMC_0-τ,ss_ is the area under the moment curve during a dosing interval |
| R_ac,AUC0-τ_ | Accumulation of AUC_0-τ_: R_ac,AUC0-τ_ = AUC_0-τ,ss_ / AUC_0-τ_ |
| R_ac,Cmax_ | Accumulation of C_max_: R_ac,Cmax_ = C_max,ss_ / C_max_ |
| AI | Accumulation index: AI = 1 / (1 - e^-λz,ss×τ^) , where τ is the dosing interval |

**Supplementary Table S6** Summary of incidence of immunogenicity

|  | **3 mg/kg**  **Q2W**  **(*n* = 10)**  ***n* (%)** | **5 mg/kg**  **Q2W**  **(*n* = 10)**  ***n* (%)** | **10 mg/kg**  **Q2W**  **(*n* = 9)**  ***n* (%)** | **20 mg/kg**  **Q2W**  **(*n* = 9)**  ***n* (%)** | **30 mg/kg**  **Q2W**  **(*n* = 5)**  ***n* (%)** | **10 mg/kg**  **Q3W**  **(*n* = 6)**  ***n* (%)** | **20 mg/kg**  **Q3W**  **(*n* = 6)**  ***n* (%)** | **Total**  **(*n* = 55)**  ***n* (%)** |
| --- | --- | --- | --- | --- | --- | --- | --- | --- |
| **Baseline** |  |  |  |  |  |  |  |  |
| **Negative** | 8 (80.0) | 9 (90.0) | 9 (100.0) | 9 (100.0) | 5 (100.0) | 6 (100.0) | 4 (66.7) | 50 (90.9) |
| **Positive** | 2 (20.0) | 1 (10.0) | 0 | 0 | 0 | 0 | 2 (33.3) | 5 (9.1) |
|  |  |  |  |  |  |  |  |  |
| **Post-baseline** |  |  |  |  |  |  |  |  |
| **Negative** | 6 (60.0) | 8 (80.0) | 9 (100.0) | 9 (100.0) | 5 (100.0) | 6 (100.0) | 4 (66.7) | 47 (85.5) |
| **Positive** | 4 (40.0) | 2 (20.0) | 0 | 0 | 0 | 0 | 2 (33.3) | 8 (14.5) |
| **Non-treatment-emergent positive** | 2 (20.0) | 1 (10.0) | 0 | 0 | 0 | 0 | 2 (33.3) | 5 (9.1) |
| **Treatment-emergent positive** | 2 (20.0) | 1 (10.0) | 0 | 0 | 0 | 0 | 0 | 3 (5.5) |
| **NAb-PD-1 positive** | 2 (20.0) | 0 | 0 | 0 | 0 | 0 | 0 | 2 (3.6) |
| **NAb-PD-1 negative** | 0 | 1 (10.0) | 0 | 0 | 0 | 0 | 0 | 1 (1.8) |
| **NAb-VEGF positive** | 2 (20.0) | 1 (10.0) | 0 | 0 | 0 | 0 | 0 | 3 (5.5) |
| **NAb-VEGF negative** | 0 | 0 | 0 | 0 | 0 | 0 | 0 | 0 |

Non-treatment-emergent positive was determined when the baseline sample was ADA-positive and the titer of any post-baseline ADA-positive sample was < 4 times the baseline ADA titer. Treatment-emergent positive was determined when the baseline sample was ADA-negative and at least one post-baseline sample was ADA-positive, or when the baseline sample was ADA-positive and at least one post-baseline sample was ADA-positive with titer ≥ 4 times the baseline ADA titer, or when the baseline sample was ADA-positive with missing baseline or post-baseline ADA titer.

PD-1, programmed death-1; VEGF, vascular endothelial growth factor; Q2W, every 2 weeks; Q3W, every 3 weeks.

**Supplementary Table S7** Serum trough concentrations of ivonescimab following multiple-dose intravenous administration

| **Sampling time point** | **Serum trough concentration (μg/mL)** | | | | | | |
| --- | --- | --- | --- | --- | --- | --- | --- |
|  | **3 mg/kg Q2W**  **(*n* = 10)** | **5 mg/kg Q2W**  **(*n* = 10)** | **10 mg/kg Q2W**  **(*n* = 9)** | **20 mg/kg Q2W**  **(*n* = 12)** | **30 mg/kg Q2W**  **(*n* = 6)** | **10 mg/kg Q3W**  **(*n* = 6)** | **20 mg/kg Q3W**  **(*n* = 6)** |
| Day 1 predose | 0.00 (0.00) | 0.00 (0.00) | 0.002 (0.0058) | 0.002 (0.0052) | 0.007 (0.0164) | 0.00 (0.00) | 0.00 (0.00) |
| Day 15 predose | 8.31 (1.67) | 11.8 (6.85) | 29.2 (7.34) | 69.6 (23.7) | 105 (20.7) | - | - |
| Day 22 predose | - | - | - | - | - | 22.0 (6.09) | 48.0 (15.5) |
| Day 29 predose | 10.3 (2.74) | 15.8 (11.8) | 40.2 (13.0) | 105 (34.9) | 142 (31.3) | - | - |
| Day 43 predose | 12.2 (2.60) | 16.2 (12.4) | 36.2 (11.1) | 120 (28.8) | 171 (46.6) | 24.2 (9.49) | 58.1 (20.5) |
| Day 57 predose | 11.3 (4.19) | 25.6 (8.58) | 43.9 (10.0) | 150 (49.9) | 193 (62.4) | - | - |
| Day 64 predose | - | - | - | - | - | 35.7 (-) | 76.8 (14.1) |
| Day 71 predose | 11.9 (4.60) | 28.2 (8.63) | 39.6 (10.7) | 142 (27.1) | 178 (53.3) | - | - |
| Day 85 predose | 12.6 (4.68) | 30.4 (9.27) | 46.4 (11.1) | 114 (38.0) | 181 (48.0) | 35.0 (-) | 63.7 (28.3) |
| Day 106 predose | - | - | - | - | - | 33.9 (-) | 76.9 (18.0) |
| Day 113 predose | 10.9 (3.25) | 32.7 (13.4) | 47.6 (13.7) | 152 (27.5) | 210 (30.3) | - | - |
| Day 141 predose | 13.1 (5.79) | 26.3 (11.1) | 51.4 (16.6) | 190 (37.5) | 185 (38.5) | - | - |
| Day 148 predose | - | - | - | - | - | 42.5 (-) | 67.4 (7.07) |
| Day 190 predose | - | - | - | - | - | 34.0 (-) | 66.4 (-) |
| Day 197 predose | 13.1 (6.71) | 29.4 (4.10) | 39.9 (2.75) | 236 (31.8) | 141 (84.0) | - | - |
| Day 232 predose | - | - | - | - | - | 38.9 (-) | - |
| Day 253 predose | 16.1 (3.11) | 31.0 (1.63) | - | 33.7 (-) | 187 (38.9) | - | - |
| Day 309 predose | 15.4 (-) | 33.1 (-) | - | - | 174 (-) | - | - |
| Day 365 predose | - | 0.016 (-)^a^ | - | - | 234 (-) | - | - |
| Day 421 predose | - | 0.112 (-)^b^ | - | - | 221 (-) | - | - |
| Day 477 predose | - | 20.4 (-) | - | - | - | - | - |

^a^The concentration of 0.016 μg/mL at Day 365 predose was from subject 10030002, and such low concentration was attributed to the fact that the patient’s last 5 visits were absent.

^b^The concentration of 0.112 μg/mL at Day 421 predose was from subject 10040011, whose low concentration was attributed to delayed administration, with a time interval of 81 days from the last administration.

Data were expressed as mean (standard deviation). Q2W, every 2 weeks; Q3W, every 3 weeks.

**Supplementary Table S8** Summary of duration of exposure to ivonescimab

| **Duration of exposure (days)** | **3 mg/kg**  **Q2W**  **(n = 10)** | **5 mg/kg**  **Q2W**  **(n = 10)** | **10 mg/kg**  **Q2W**  **(n = 9)** | **20 mg/kg**  **Q2W**  **(n = 9)** | **30 mg/kg Q2W**  **(n = 5)** | **10 mg/kg Q3W**  **(n = 6)** | **20 mg/kg Q3W**  **(n = 6)** | **Total**  **(n = 55)** |
| --- | --- | --- | --- | --- | --- | --- | --- | --- |
| Mean (SD) | 138.0  (109.76) | 178.6  (184.90) | 130.6  (102.28) | 147.6  (180.69) | 277.0  (238.93) | 96.2  (86.29) | 141.8  (64.98) | 156.0  (151.33) |
| Median (Q1, Q3 ) | 87.5  (57.0, 224.0) | 113.0  (43.0, 203.0) | 114.0  (42.0, 224.0) | 81.0  (42.0, 172.0) | 237.5  (118.0, 325.0) | 64.0  (62.0, 74.0) | 137.0  (115.0, 189.0) | 113.0  (56.0, 224.0) |
| Min, Max | 56, 336 | 43, 584 | 14, 278 | 28, 664 | 28, 716 | 42, 271 | 42, 231 | 14, 716 |


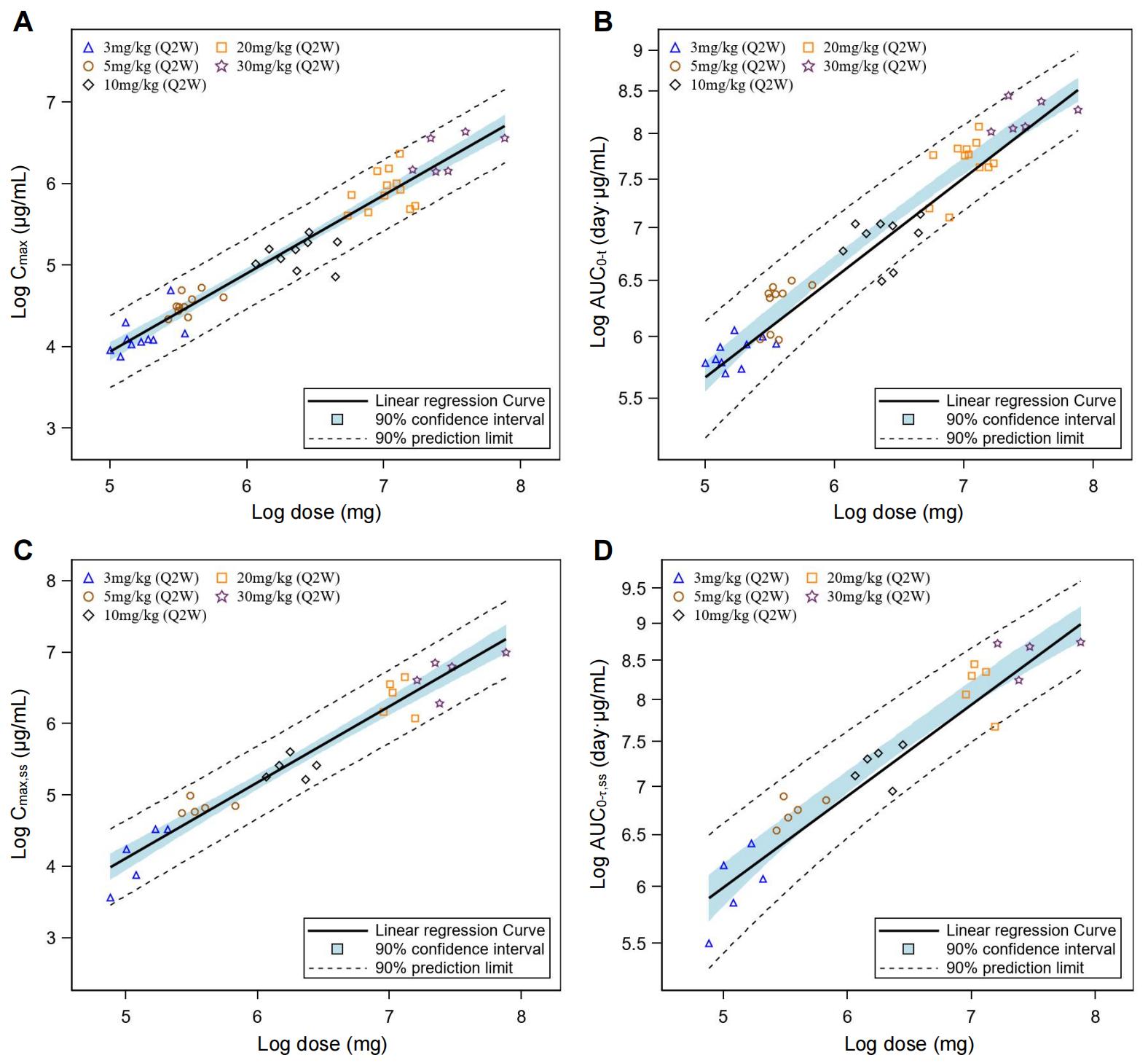


**Supplementary Figure S1** Relationship between dose and pharmacokinetic parameters of ivonescimab. (A) Profiles of log C_max_ versus log dose and (B) log AUC_0-t_ versus log dose after a single dose ranging 3-30 mg/kg. (C) Profiles of log C_max,ss_ versus log dose and (D) log AUC_0-τ,ss_ versus log dose after multiple doses ranging 3-30 mg/kg. The empty shapes represent individual observed values, the shaded areas represent 90% confidence intervals, the solid lines are the linear regression curves, and the dashed lines are the 90% prediction limits. C_max_, the maximum concentration; AUC_0-t_, the area under the concentration-time curve from 0 to t days; C_max,ss_, the maximum concentration at steady state; AUC_0-τ,ss_, the area under the concentration-time curve during a dosing interval at steady state; Q2W, every 2 weeks.
